# Micrometeorite collections: a review and their current status

**DOI:** 10.1098/rsta.2023.0195

**Published:** 2024-06-23

**Authors:** Matthias van Ginneken, Penelope J. Wozniakiewicz, Donald E. Brownlee, Vinciane Debaille, Vincenzo Della Corte, Lucie Delauche, Jean Duprat, Cecile Engrand, Luigi Folco, Marc Fries, Jérôme Gattacceca, Matthew J. Genge, Steven Goderis, Matthieu Gounelle, Ralph P. Harvey, Guido Jonker, Lisa Krämer Ruggiu, Jon Larsen, James H. Lever, Takaaki Noguchi, Scott Peterson, Pierre Rochette, Julien Rojas, Alessandra Rotundi, N. G. Rudraswami, Martin D. Suttle, Susan Taylor, Flore Van Maldeghem, Michael Zolensky

**Affiliations:** ^1^ Centre for Astrophysics and Planetary Science, School of Physics and Astronomy, University of Kent, Canterbury CT2 7NH, UK; ^2^ Department of Astronomy, University of Washington, Seattle, WA 98195, USA; ^3^ Laboratoire G-Time, Université Libre de Bruxelles, Brussels 1050, Belgium; ^4^ Istituto di Astrofisica e Planetologia Spaziali—INAF, Roma, Italy; ^5^ IJCLab, Université Paris-Saclay, CNRS/IN2P3, Orsay 91405, France; ^6^ IMPMC, CNRS-MNHN-Sorbonne Universités, UMR7590, 57 rue Cuvier, Paris 75005, France; ^7^ Dipartimento di Scienze della Terra, Università di Pisa, Pisa, Italy; ^8^ NASA Johnson Space Center, Astromaterials Research and Exploration Science (ARES), Houston, TX 77058, USA; ^9^ CEREGE, CNRS, Aix-Marseille Univ, IRD, INRAE, Aix-en-Provence, France; ^10^ Impacts and Astromaterials Research Centre, Department of Earth Science and Engineering, Imperial College London, , UK; ^11^ Archaeology, Environmental Changes and Geo-Chemistry, Vrije Universiteit Brussel, Brussels BE 1050, Belgium; ^12^ Department of Geological Sciences, 112 A. W. Smith Building, Case Western Reserve University, Cleveland, OH 44106-7216, USA; ^13^ Faculty of Science, Department of Earth Sciences, Vrije Universiteit Amsterdam, Amsterdam, Netherlands; ^14^ Project Stardust, Oslo, Norway; ^15^ Cold Regions Research and Engineering Laboratory, Hanover, NH, USA; ^16^ Division of Earth and Planetary Sciences, Kyoto University, Kyoto 606-8502, Japan; ^17^ Citizen Scientist, Minneapolis, MN, USA; ^18^ Earth and Planets Laboratory, Carnegie Institution of Washington, Washington, DC 20015, USA; ^19^ Dipartimento di Scienze Applicate, Universita` degli Studi di Napoli ‘‘Parthenope’’, Napoli, Italy; ^20^ CSIR-National Institute of Oceanography, Goa 403 004, India; ^21^ School of Physical Sciences, The Open University, Milton Keynes MK7 6AA, UK

**Keywords:** micrometeorites, cosmic dust, planetary science

## Abstract

Micrometeorites are estimated to represent the main part of the present flux of extraterrestrial matter found on the Earth’s surface and provide valuable samples to probe the interplanetary medium. Here, we describe large and representative collections of micrometeorites currently available to the scientific community. These include Antarctic collections from surface ice and snow, as well as glacial sediments from the eroded top of nunataks—summits outcropping from the icesheet—and moraines. Collections extracted from deep-sea sediments (DSS) produced a large number of micrometeorites, in particular, iron-rich cosmic spherules that are rarer in other collections. Collections from the old and stable surface of the Atacama Desert show that finding large numbers of micrometeorites is not restricted to polar regions or DSS. The advent of rooftop collections marks an important step into involving citizen science in the study of micrometeorites, as well as providing potential sampling locations over all latitudes to explore the modern flux. We explore their strengths of the collections to address specific scientific questions and their potential weaknesses. The future of micrometeorite research will involve the finding of large fossil micrometeorite collections and benefit from recent advances in sampling cosmic dust directly from the air.

This article is part of the theme issue ‘Dust in the Solar System and beyond’.

## 1. Introduction

Every year Earth accretes 20 000 to 40 000 tonnes of cosmic dust, of which approximately 10% survive atmospheric entry to become micrometeorites. This represents in terms of mass the largest part of the flux of extraterrestrial material reaching the Earth’s surface [1]. The sizes of micrometeorites range from a few tens to 2000 µm [2]. Cosmic dust particles are <10 µm that are typically collected in the stratosphere and are termed interplanetary dust particles (IDPs) [3]. Micrometeorites originate from small primitive parent bodies (i.e. non-differentiated); for most individual particles deciphering between asteroidal and cometary sources remains ambiguous however contribution from comets may dominate the lower size range (<50 µm) [4] while the contribution from asteroids may dominate above this threshold [5]. This is in sharp contrast with models suggesting that the zodiacal cloud, which includes micrometeoroids, is mostly constituted of cometary dust, particularly from Jupiter Family Comets [6]. Recent studies also suggest that micrometeorites may sample isotopic reservoirs not observed in meteorite collections [7]. Therefore, micrometeorites are an essential tool for probing the interplanetary medium and estimating the input of extraterrestrial matter to the geochemical budget of Earth. The continuous flux of micrometeorites to Earth allows their collection on any surface, provided the accumulation time is sufficient and environmental conditions allow their preservation. The extraterrestrial nature of micrometeorites was first determined by the finding and chemical analysis of dark magnetic spheres in deep-sea sediments (DSS) collected during the 1872–1876 expedition of the HMS Challenger [8]. Since then, large numbers of micrometeorites have been sampled from various environments, mainly from DSS, ice or snow and glacial sediments from polar regions. Micrometeorites from DSS were collected by raking, dredging and magnetically extracting cosmic spherules [9–14]. In polar regions, micrometeorites were first collected in Greenland ice [15], and subsequently in Greenland cryoconite [16], Antarctic ice [17,18] or glacial deposits [19,20].

More recent collections have focussed on the systematic sampling of unbiased or rare micrometeorite populations. This paper reviews the status of these major micrometeorite collections, including their statistical properties and scientific potential. The strengths and possible biases of the collections are discussed to estimate their potential to address specific scientific problems. Finally, future prospects for the collection of micrometeorites are explored.

## 2. The classification of micrometeorites

**Figure 1.**
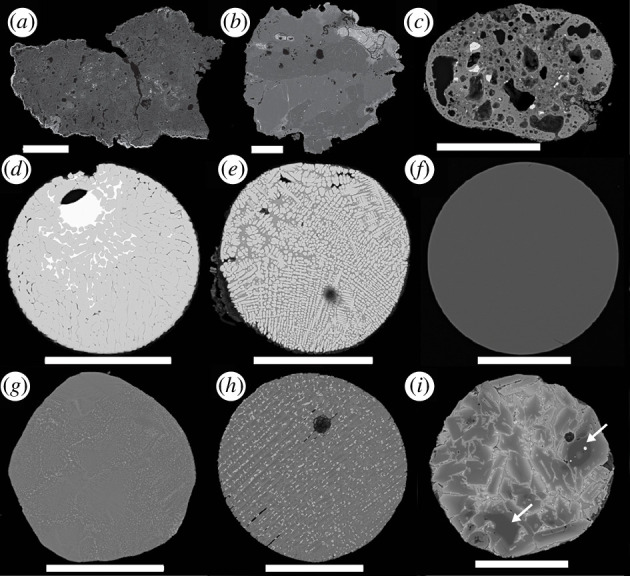
Scanning electron backscatter images of sectioned micrometeorites from the Transantarctic Mountains (*a*,*b*) and Larkman Nunatak (*c*–*i*) collections. (*a*) A fine-grained unmelted micrometeorite. (*b*) A coarse-grained unmelted micrometeorite. (*c*) A scoriaceous micrometeorite. (*d*) A I-type cosmic spherule. (*e*) A G-type cosmic spherule. (*f*) A V-type cosmic spherule. (*g*) A CC cosmic spherule. (*h*) A barred olivine (BO) cosmic spherule. (*i*) A porphyritic olivine (Po) cosmic spherule. Arrows indicate relict olivine. Scale bars 100 µm.

**Figure 2.**
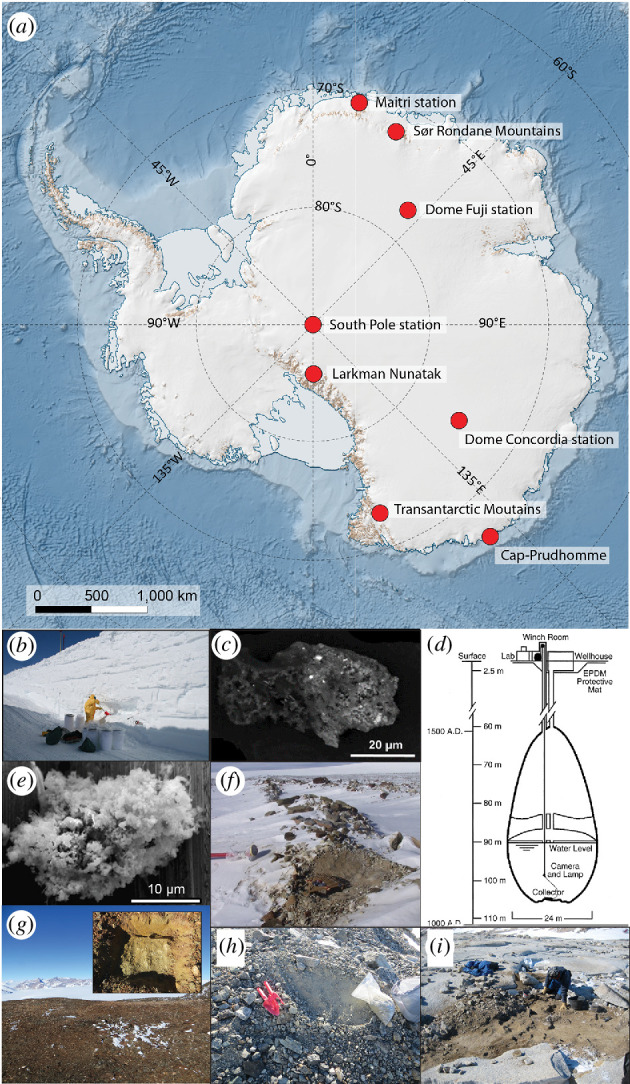
Sampling locations of Antarctic collections (*a*). (*b*) Snow sampling in a trench near Dome C. Photo credit: J. Duprat, MNHN. (*c*) Backscatter electron image of an UCAMM from Dome C [32]. (*d*) Approximate size and shape of the South Pole water well (SPWW) in December 1995. (*e*) Secondary electron image of CP micrometeorite D07IB39 [33]. (*f*) Detail of the sampling area at Larkman Nunatak. Photo credit: Matthew Genge, ICL. (*g*) Glacially eroded surface of Walnumfjellet in the Sør Rondane Mountains (inset shows the 30 cm × 30 cm × 6 cm sampling area). (*h*) Sampling location on a moraine near Wideroefjellet in the Sør Rondane Mountains. Photo credit for (*g,h*): Matthias van Ginneken, UKC. (*i*) Sampling of glacial sediment on a flat eroded summit of the Transantarctic Mountains. Photo credit: Luigi Folco, UniPi. The map was made using the Quantarctica package for QGIS [34].

Micrometeorites are classified on the basis of the degree of thermal alteration suffered during atmospheric entry into three groups, the unmelted, partially melted and melted micrometeorites [21]. Unmelted micrometeorites, typically angular to subangular particles, are classified primarily into the fined-grained, coarse-grained and ultracarbonaceous subtypes based on their main constituent mineral phases. The fine-grained (Fg) micrometeorites are similar to the matrices of certain carbonaceous chondrites and often exhibit evidence of limited thermal alteration [22–25] (figure 1*a*). The coarse-grained (Cg) variants are dominated by anhydrous chondritic minerals and with textures suggestive of ordinary chondrites, chondrules or even differentiated meteorites [26–28] (figure 1*b*). Ultracarbonaceous-type micrometeorites (UCAMMs), discovered in Antarctic snow, are dominated by organic matter and consist of fine-grained, porous aggregates of anhydrous mafic silicate crystals within networks of highly disorganized carbon [29–31] (figure 2*c*). More recently, chondritic porous (CP) micrometeorites indistinguishable from CP IDPs were discovered in Antarctic snow [33] (figure 2*e*).

Partially melted micrometeorites are mostly subrounded silicate particles with a scoriaceous texture and are often mantled by a magnetite rim due to the oxidation of iron during atmospheric entry heating [35] (figure 1*c*). They contain relict grains of forsterite and/or enstatite, enveloped by an igneous mesostasis formed during atmospheric entry, featuring microphenocrysts of fayalitic olivine and magnetite in vesicular silicate glass [36,37].

Melted micrometeorites are typically spherical owing to surface tension upon melting and are usually qualified as cosmic spherules. These are further subdivided into iron-rich (I-type; figure 1*d*), glass with magnetite (G-type; figure 1*e*) and silicate-type (S-type) cosmic spherules, each exhibiting unique compositions, textures and mineralogy. I-type spherules are dominated by magnetite and wüstite [38]. G-type spherules are characterized by magnetite dendrites within silicate glass and are intermediate in composition between I-type and S-type. S-type cosmic spherules, primarily silicate-based and the most prevalent, exhibit a variety of subclasses, each reflecting different atmospheric entry conditions. CAT spherules, enriched in Ca, Al and Ti with high Mg/Si ratios with respect to other S-type spherules, formed following the highest peak temperatures and undergo rapid weathering. Glass CSs, or V-type, are transparent and often vesiculated, form at high temperatures and may contain Fe–Ni metal beads (figure 1*f*). Cryptocrystalline (CC) spherules, featuring submicron crystallites and magnetite, experienced slightly lower peak temperatures (figure 1*g*). BO spherules, with parallel growth olivine, form at temperatures lower than CC spherules (figure 1*h*). Lastly, Po spherules, marked by olivine microphenocrysts within a glassy mesostasis, endure the lowest peak temperatures, especially those containing relict minerals (figure 1*i*).

## 3. Major collections

The systematic sampling of micrometeorites largely coincides with improvements in analytical techniques allowing the characterization of microscopic geological samples in the early 1980s [14,16,39]. In addition to the development of new sampling methods, this allowed the establishment of large collections containing hundreds to tens of thousands of samples. The criteria used here to consider collections major includes their size, maximum accumulation age, availability to the scientific community or presence of rare types of micrometeorites. Basic information and statistics on major collections are reported in table 1.

**Table 1.** Main properties and statistics of the micrometeorite collections.

collection	Cap Prudhomme	Concordia	Dome Fuji	Maitri	South Pole Water Well	Larkman Nunatak	Sør Rondane Mountain	Transantarctic Mountains^a^	Indian Ocean	Atacama Desert	Budel	Jon Larsen	Scott Peterson
*sampling location*	*Antarctica*	*Antarctica*	*Antarctica*	*Antarctica*	*Antarctica*	*Antarctica*	*Antarctica*	*Antarctica*	*Indian Ocean*	*Atacama Desert, Chile*	*Netherlands*	*International (several rooftops*)	*USA (several rooftops*)
type	ice	snow	snow	ice	ice	Moraine	weathering traps	weathering traps	deep-sea sediment	desert soil	urban (single rooftop)	urban	urbans
status of MM extraction	ongoing	ongoing	ongoing	ongoing	complete	ongoing	ongoing	ongoing	ongoing	ongoing	complete	ongoing	ongoing
curation facility (institution, country)	currently IJCLab, Future : MNHN, France	currently IJCLab, Future : MNHN, France	Kyoto University, Japan	National Institute of Oceanography, India	Johnson Space Center, USA	Imperial College London, UK	Vrije Universiteit Brussel, Belgium	Università di Pisa, Italy	National Institute of Oceanography, India	CEREGE, France	Vrije Universiteit Amsterdam, Netherlands	private	private
extraction method	pumping and melt water filtering	melt water filtering	melt water filtering	melt water filtering + magnetic	suctioning and melt water filtering	manual (magnetic and non-magnetic)	manual (magnetic and non-magnetic)	manual	manual (magnetic)	manual (magnetic + non-magnetic)	shape + density + manual	manual (magnetic)	manual (magnetic)
MM accumulation time window	<50 000 years	20 years (1950–1970)	<2 years	N/A	~700 years	~800 ka	>1 Ma	≥2.3 Ma	~50 000 years	>1 Myr	3.5 years (2018–2022)	<50 years	<50 years
size fraction investigated (μm)	>25	>20	<10–200	>50	50–2000	50–800	20–2000	60–300	50–300	200–850	80–515	150–1000	70–900
size distribution: slope value					−5.2 ± 0.5^b^	−5.3^c^	4.8 to −5.2^d^	−4.8^a^	−3.9^e^	−5.5	−4.88^f^		
size distribution: range fitted (μm)					200–500	210−330	200–800	200–400	200–500	200–400	180–450		
No. of MMs extracted and/or prepared	>10 000	3009	1025	2986	>7000	1450	20 761	3468	1886	789	1006	4714	4012
estimated total number of MMs	>100 000	>10 000		>7000	>>10 000		>>10 000	>100 000	>>20 000	>>2000			
number of unmelted MMs	N/A	1915	843	N/A	755	17	1080	75	195	N/A	N/A	16	19
fine-grained	N/A	1140	N/A	N/A	511	11	918	32	N/A	N/A	N/A	N/A	N/A
coarse-grained	N/A	247	N/A	N/A	227	6	162	31	N/A	N/A	N/A	N/A	N/A
refractory	N/A	4	2	N/A	17	N/A	N/A	13	N/A	N/A	N/A	N/A	N/A
UCAMM	N/A	24	3	N/A	N/A	N/A	N/A	N/A	N/A	N/A	N/A	N/A	N/A
CP IDP-like	N/A	312	30	N/A	N/A	N/A	N/A	N/A	N/A	N/A	N/A	N/A	N/A
number of scoriaceous MMs	N/A	500	51	133	1364 (including Po CSs)	16	685	164	38	N/A	26	48	61
number of cosmic spherules	N/A	1094	131	2852	3565	1355	18 996	3229	1653	789	980	4650	3932
CSs I-types	N/A	N/A	N/A	181	48	124	627	147	183	129 (including G-type)	N/A	2	6
CSs G-types	N/A	N/A	N/A	37	50	28	323	152	45	129 (including I-type)	N/A	2	3
CSs S-types	N/A	N/A	N/A	2634	3467	1200	18 046	2930	1425	667	980	4646	3923
CSs S-types: Po	N/A	N/A	N/A	1190	1364 (including Scoriaceous MMs)	425	1805	404	388	N/A	385	1140	619
CSs S-types: BO	N/A	N/A	N/A	1090	1307	286	5604	611	600	N/A	264	2313	2380
CSs S-types: CC	N/A	N/A	N/A	221	455	255	3970	1571	275	N/A	295	1111	704
CSs S-types: V	N/A	N/A	N/A	133	1648	226	6687	230	162	N/A	36	82	220
CSs S-types: CAT	N/A	N/A	N/A	N/A	57	N/A	3641	N/A	N/A	N/A	N/A	N/A	N/A

^a^ Statistics from micrometeorite trap TAM65 only [40].

^b^ From [41].

^c^ From [42].

^d^ From [43].

^e^ From [44].

^f^ From [45].

MM, micrometeorite; N/A, not available..

### 3.1. Antarctic collections

The Antarctic environment provides ideal conditions for the accumulation, preservation and identification of extraterrestrial materials, such as meteorites and micrometeorites [37,46–48]. As a result, the largest and least biased collections of micrometeorites originate from Antarctica. The locations of the Antarctic collections listed here are shown in figure 2*a*. Below we describe the sampling processes and basic inventories of modern Antarctic micrometeorite collections.

#### 3.1.1. Snow and ice collections

##### 3.1.1.1. The Cap-Prudhomme collection

From 1988 to 1998, four micrometeorite sampling campaigns took place 6 km south of the French station of Dumont d’Urville and on the Astrolabe glacier, Adélie Land, Antarctica (6°40′ S–140°01′ E; figure 2*a*) [48–50]. Ultra-clean surface ice was melted by injecting 70°C water in 2 m deep drill holes, creating pockets of melt water. About 600 tonnes of ice was melted over the years. This procedure allowed micrometeorites trapped within the ice to be released in the water to settle on the bottom of the pockets, along with terrestrial particles. This glacial deposit was then pumped to the surface and size-sorted using stainless steel sieves in four size fractions (25–50, 50–100, 100–400 and >400 µm). From the 1994 season onwards, all sampling components exposed to hot water were made of either stainless steel used in French nuclear reactor tubing or Teflon to reduce the risk of corrosion and leach product contamination in the melt water [22]. Daily collections of glacial sand were stored in vials, either glass or Teflon, with a small amount of their original melt water and kept at −20°C. Micrometeorites can be directly handpicked from the sand, with the highest proportion of extraterrestrial to terrestrial particles of about 20% in the 50–100 µm size range.

While detailed statistics on the collection are not available, about 10 000 micrometeorites have been identified over the years, making it one of the largest so far [22]. In the 50–100 size range, unmelted micrometeorites represent about 80% of the particles and ~20% are melted cosmic spherules. In the 100–400 µm size range, these proportions are inverted, with ~80% of cosmic spherules and ~20% of unmelted extraterrestrial particles [51]. The samples are mostly whole particles that are currently stored under nitrogen gas in a cleanroom facility at the Laboratoire de Physique des 2 Infinis Irène Joliot Curie (IJCLab), France. Some samples can be available upon request. Long-term curation will involve transferring the samples to the National Museum of Natural History of Paris (MNHN), France, in the future.

##### 3.1.1.2. CONCORDIA collection at Dome C

Micrometeorites were obtained from snow in the vicinity of the French-Italian CONCORDIA station at Dome C in Antarctica (75°05′ S, 123°19′ E; figure 2*a*) during six field expeditions in 2000, 2002, 2006, 2014, 2016 and 2019. The sampling location was chosen for its remoteness and major wind direction towards the edge of the continent, preventing the influx of terrestrial dust from higher latitudes [37]. Snow was manually collected near the surface in 2000 (0–80 cm depth), and from 2002, it was collected from clean trenches 3–4 m deep a few kilometres away from Dome C, to minimize the contamination (figure 2*b*). The collected snow is estimated to date back to 1970–1980, that is before human activity started at Dome C, ensuring the pristine nature of the samples. Snow samples were transferred to Dome C and melted in a double stainless steel tank melter. The water was then gently filtered and the dust was recovered on a 20 µm nylon filter. To avoid the destruction of the most friable samples, exposure to water was limited to a maximum of 20 h and no mechanical pumping was used. Potential contamination of the filters was constantly monitored during the filtering process. The filters were sealed under dry nitrogen in Antarctica, shipped back to France and opened in a cleanroom at IJClab. Micrometeorites in the 20–500 µm size range were then handpicked under a binocular microscope.

The ongoing extraction of Concordia micrometeorites has yielded 3009 extraterrestrial particles so far, which have been individually classified (table 1). Of these, 1140 were fine-grained (including the CP micrometeorites), 247 course-grained and 4 refractory micrometeorites. Significantly, the collection contains 24 UCAMMs and 312 CP micrometeorites (figure 2*c*). All classified micrometeorites were fragmented into several pieces and the samples are mostly whole particles that are currently held under nitrogen gas at IJCLab, to prevent potential effects of terrestrial alteration . Several filters from recent Antarctic expeditions await the handpicking of whole particles. Some samples can be available upon request. The Concordia collection will be transferred to the MNHN in the future.

##### 3.1.1.3. Dome Fuji collection

From 2003 to 2012, approximately 1 tonne of surface snow was sampled near the Antarctic Japanese Dome Fuji Station (77◦19′S, 39◦42′E, figure 2*a*), Antarctica, to a depth of approximately 10 cm [33,52]. Given the accumulation rate of 10 cm/yr [53], the micrometeorites were collected approximately 1 year after their deposition for samples collected in 2007, 2010 and 2012, and 2 years for samples collected in 2003 and 2005. Snow samples were then shipped to Ibaraki University via the National Institute of Polar Research (NIPR), Japan, in a frozen state, to be melted and filtered in a clean room. Finally, micrometeorites are handpicked or picked up using a micromanipulator for particles smaller than 30 µm, under a binocular microscope. As of now, this represents the youngest micrometeorite collection from Antarctica. Coupled with a maximum temperature of −30°C at Dome Fuji, this results in a virtual absence of terrestrial weathering effects observable under a scanning electron microscope (SEM) and transmission electron microscope (TEM).

To date, 1025 micrometeorites were gathered, comprising 843 unmelted, 51 scoriaceous and 131 cosmic spherules, with average sizes of 40 µm, 64 µm and 40 µm, respectively (table 1). In particular, unmelted micrometeorites include 2 refractory micrometeorites, 3 UCAMMs and 30 CP micrometeorites (figure 2*e*). Extraction of micrometeorites is ongoing and more samples are expected in the future. As of early 2024, samples from this collection are not available upon request, but a future transfer at an unknown date to the NIPR, Japan, and access to samples is envisioned in the future.

##### 3.1.1.4. Maitri collection

The Indian Maitri station, Antarctica is located (70°45′S, 11°44′E) on the Schirmacher Oasis, about ~8 km from ice fields from where the micrometeorites are collected. The collection was performed by excavating blue ice from November 2015 to February 2016 up to a depth of approximately 1 m. One tonne of ice was excavated each day of operation, from which the micrometeorites were extracted. The total weight of ice sampled during this expedition was ~50 tonnes. The collected ice was transferred to a clean vessel, melted and subsequently sieved at above 50 µm. The sieved fraction of the sample was dried, before carrying out a magnetic separation. Micrometeorites were then handpicked under a binocular microscope.

Total number of micrometeorites extracted so far is 2986, including 133 scoriaceous micrometeorites and 2852 cosmic spherules (table 1). Of these cosmic spherules, 181 are I-type, 37 are G-type and 2634 are S-type (see table 1 for details on the inventory of the subtypes of S-type cosmic spherules). The extraction of micrometeorites is ongoing at the National Institute of Oceanography (NIO), India, where the samples are curated and available upon request.

##### 3.1.1.5. South Pole water well collection

The South Pole drinking water well (SPWW) at the US Amundsen-Scott South Pole Station (90°S, 0°E; figure 2*a*), slowly and continuously melted pre-industrial ice. Warm water introduced 10 m above the well bottom, produced laminar flow and bulk water temperatures less than 4°C. The well melted downward at a rate of ~2 cm/d yielding a smooth, though sculptured and fracture-free ice bottom [54]. Circulation velocities were insufficient to entrain the micrometeorites of interest (> 50 µm) thereby concentrating the particles on the bottom as a lag deposit. A remotely controlled collector suctioned and internally filtered particles from the ice surface while traversing the well bottom [55]. A depth-to-age relationship obtained from a South Pole ice core [56] gave a good age constraint for the collected samples (figure 2*d*). In December 1995, the well had a volume of 5000 m^3^ and was 106 m deep corresponding to an age interval of 1100–1500 AD. An area of 30 m^2^ of the well bottom was vacuumed and ~200 g of material was retrieved of which about 0.1% were (melted) micrometeorites. At that time, 1883 micrometeorites were handpicked, mounted embedded in Epoxy resin and sized. In November 2000, the well had a volume of 4000 m^3^, and was 134 m deep corresponding to an age interval of 800–1100 AD [56]. The central plateau was suctioned, as well as all the surrounding pockets [57]. The 11 deployments yielded about 40 g of material. Hand sorting of several different samples suggested that about 1% of the collected material was extraterrestrial. Compared with the 1995 collection, the 2000 samples had less iron oxide (a contaminant from the well’s pump failure in 1994) allowing the recovery of many angular unmelted micrometeorites.

Of the 5682 micrometeorites classified, 9% were fine-grained unmelted, 4% coarse-grained unmelted, 6% scoriaceous, 3% relict grain bearing, 15% porphyritic cosmic spherules, 23% BO cosmic spherules, 8% CC cosmic spherules, 29% V-type cosmic spherules, 1% CAT cosmic spherules, 1% G-type cosmic spherules, 1% I-type cosmic spherules and 0.3% single mineral micrometeorites. Rare types include seven micrometeorites with major and minor element compositions consistent with values typical of basaltic, howardite, eucrite and diogenite (HED) meteorites [58,59], 1 micrometeorite that is likely lunar [60] and 1 micrometeorite that is probably a calcium aluminium inclusion [61]. The extraction of the micrometeorites is now complete and all of the SPWW samples reside at Johnson Space Center in Houston and can be requested from the Astromaterials Research and Exploration Science Division (https://curator.jsc.nasa.gov/dust/).

### 3.1.2. Glacial sediment collections

Here, we use the term glacial sediment to describe the collections extracted from moraine deposits and fined-grained detritus found in weathering pits and cracks present on the often glacially eroded tops of nunataks in Antarctica.

#### 3.1.2.1. Larkman Nunatak collection

The sampling site, Larkman Nunatak (86°46′S, 179°20′E; figure 2*a*), Antarctica, consists of a main nunatak and two smaller satellite outcrops, with exposed basalt outcrops on multiple sides, raised ice pressure ridges, wind scoops and an area of meteorite accumulation [42]. About 3 kg of fine-grained material were collected from supraglacial moraines near Larkman Nunatak at the boundary between blue ice and the overlying snow (figure 2*f*). The discovery of Australasian microtektites in the sample suggests that the micrometeorite accumulation period covers the last 800 kyr at least [62]. Snow was removed using a scoop and the fine-grained sample, which included the full depth of the sediment deposit, was placed into a sealed plastic bag. Care was taken to ensure no meteorites or snow was accidentally included with the sample. For the micrometeorite sampling process, dry separates of moraine fines from the site were initially examined under a binocular microscope to assess the abundance of cosmic spherules. Micrometeorites meeting the criteria established in [21] were handpicked under a binocular microscope. Some samples underwent magnetic separation techniques to concentrate micrometeorites, resulting in a magnetic fraction comprising 1.7% of the deposit, which contained terrestrial magnetite, ilmenite, basalt particles, magnetite-bearing sedimentary particles and micrometeorites. Approximately 70% of the cosmic spherules were subjected to ultrasound cleaning with a water mixed with hydrogen peroxide to remove micron-sized dust particles and mineral encrustations.

The number of characterized micrometeorites currently amounts to 1450, including 17 unmelted micrometeorites, 16 scoriaceous micrometeorites and 1355 cosmic spherules (table 1). Unmelted micrometeorites comprise 11 fine-grained and 7 coarse-grained. Of the 1355 cosmic spherules, 124 are I-types, 28 G-types and 1200 S-types. Within the S-types, subtypes consist of 425 Po, 286 BO, 255 CC and 226 V-type. No CAT S-type cosmic spherules were identified in this collection. The classified micrometeorites are currently kept under ambient environmental conditions at the School of Earth Science and Engineering of Imperial College London, UK. As of early 2024, the samples are not available upon request but may be in the future.

#### 3.1.2.2. Sør Rondane mountains collection

The Sør Rondane Mountain range is situated within Dronning Maud Land of East Antarctica and covers a surface area of approximately 2000 km^2^. During the 2012–2013 BELAM (Belgian Antarctic Meteorites), about 6.6 kg of fine-grained glacial sediment was sampled from weathering cracks near the summit of Widerøefjellet (2409 m.a.s.l.; 72°8′41″ S, 23°16′41″ E; figure 2*a*) [63] as well as other mountain summits. This site was sampled again during the 2017–2018 BELAM expedition, along with about 20 other sites, including the flat glacially eroded top of Walnumfjellet (1567–2489 m.a.s.l.; 72°07′11″ S, 24°12′30″ E; figure 2*g*) and moraines (figure 2*h*), totalling over 100 kg of glacial sediment. In 2020 and 2023, smaller sediment volumes were sampled again from several of these sites and new sites in the area.

Exposure ages of the sampling areas were determined using cosmogenic nuclides and suggested that the sampling locations accumulated micrometeorites for at least 1 Myr [64,65]. Samples were typically collected using non-metallic shovels, brushes and polyethylene sample bags. Rocks larger than 1 cm were removed onsite using a 2 mm meshed sieve. When present, ice in samples from supraglacial moraines were melted and dried at 60°C in a vacuum oven immediately after sampling at the Belgian Princess Elisabeth Station. Samples were then shipped to Vrije Universiteit Brussel (VUB) in Belgium and subsequently dry- or wet-sieved using the following mesh sizes: 2000—800—400—200—100 µm. Micrometeorites were handpicked under a binocular microscope. To extract the usually non-magnetic V-type cosmic spherules, magnetic extraction was avoided when possible.

As of now, 20 761 micrometeorites have been extracted, including approximately 1080 unmelted micrometeorites (~918 Fg and ~162 Cg), ~685 scoriaceous micrometeorites and ~18 996 cosmic spherules based on smaller aliquots studied in detail that were sectioned and studied using a SEM. I-type and G-type cosmic spherules account for ~627 and ~323, respectively, while S-type is ~18 046 (1805 Po; 5604 BO; 3970 CC; 6687 V-type; and 3799 CAT). Australasian microtektites and spherules resulting from a large airburst 430 kyr ago are also part of this collection [65,66]. Extraction of micrometeorites is ongoing. The concentration of micrometeorites and the quantity of sample that has yet to be explored suggest that there are between 30 000 and 50 000 micrometeorites in the remaining fractions of the collected materials. The samples are currently held under low humidity conditions in desiccators and are available upon request from the Archaeology, Environmental Changes & Geo-Chemistry Department of the VUB.

#### 3.1.2.3. Transantarctic mountains collection

Large accumulations of micrometeorites were discovered by an Italian National Antarctic Research Program (PNRA) team on the Myr-old summits of several isolated nunataks in the Victoria Land Transantarctic Mountains (TAM) during the 2003 austral summer [67] (figure 2*a*). The long micrometeorite accumulation period is based on old exposure age of the sampled surfaces [68] and the presence of 800 kyr old Australasian microtektites [69]. Since then, several collection campaigns have been undertaken by PNRA teams which led to the recovery of tens of thousands of micrometeorites. The samples of glacial sediments were collected from weathering pits and cracks present on the flat eroded summits of nunataks (figure 2*i*). Samples were first dried using vacuum pumping and dry-sieved into several size fractions (100–200, 200–400, 400–800, 800–2000 and >2000 μm). Micrometeorites were then magnetically and visually extracted under a binocular microscope. More recent sampling involved magnetic extraction directly on the field. The number of micrometeorites reported after the first expedition amounted to 160 unmelted micrometeorites and 6702 cosmic spherules, including 47 and 96 larger than >800 µm, respectively [67]. This high number of ‘giant’ (>400 µm) micrometeorites is unique to this collection [26].

The following statistics, which are also reported in table 1, are from a representative single sampling location (TAM65) on the summit plateau of the Miller Butte nunatak (72°42′03.30″ S, 160°15′34.20″ E), Victoria Land Transantarctic Mountains (figure 1*i*), during the 2017–2018 PNRA expedition [40]. About 15 kg of glacial sediment was sampled, of which only a fraction was explored.

The number of 3468 micrometeorites extracted from TAM65 is taken as a reference here [40] and represents only a fraction of the actual total number in this collection. This comprises 76 unmelted micrometeorites, 164 scoriaceous micrometeorites and 3229 cosmic spherules. Fine-grained and coarse-grained unmelted micrometeorites amount to 32 and 31, respectively, along with 13 refractory micrometeorites. Cosmic spherules can be subdivided into 147 I-types, 152 G-types and 2930 S-types, of which the subtypes are 404 Po, 611 BO, 1571 CC and 230 V-types. Note that while CAT spherules were identified in the TAM collection, they were not in this aliquot from TAM65. The collection also includes hundreds of Australasian microtektites as well as unique aggregates of thousands of spherules smaller than 100 µm that formed during a Tunguska-like airburst about 480 ky ago [69–71]. It is estimated the TAM collection contains >1 00 000 micrometeorites, for which full classification statistics are not yet available. The micrometeorites are maintained under ambient environmental conditions and are available upon request at the Museo Nazionale dell’Antartide in Siena, Italy.

### 3.2. Deep-sea collections

#### 3.2.1. The DSSs collection

Beyond their scientific values, DSS collections are of historical significance as they represent the first identification of cosmic spherules during the 1872–1876 expedition of the HMS Challenger [8]. Microscope slides on which the micrometeorites were stored are part of the meteorite collection of the Natural History Museum in London, UK. Furthermore, this environment was the target of the first attempts at large-scale sampling of micrometeorites, notably by using magnetic rakes that were dragged across the ocean floor 1000 km east of Hawaii to extract large numbers of cosmic spherules [11,14,39,72]. Twenty mounts containing 1572 micrometeorites from the DSS collection are currently curated and available upon request at the Johnson Space Center in Houston.

#### 3.2.2. Indian Ocean collection

**Figure 3.**
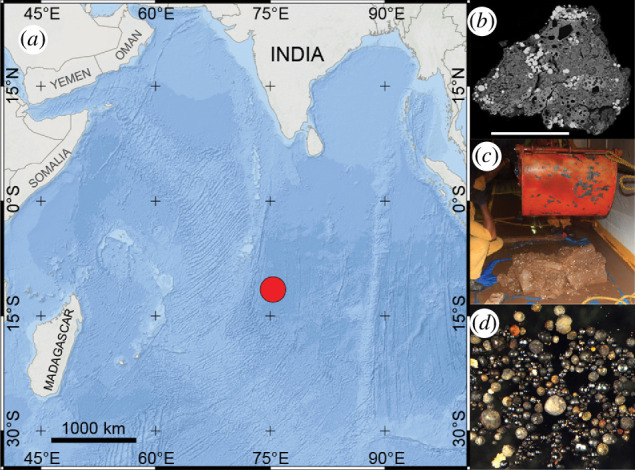
Geographical area of DSS was sampled in the Indian Ocean (*a*). (*b*) Electron backscattered image of a sectioned unmelted micrometeorite from the DSS collection. Scalebar is 100 µm. (*c*) Result of a Grab Sampler operation showing a large quantity of DSS from the Indian Ocean. (*d*) Spherules were extracted from the DSS on the research vessel shortly after sampling in the Indian Ocean. Photo credit: NIO members.

Nearly 5 tonnes of DSSs were collected from central Indian Ocean using Grab sampler from several locations in an area measuring 150 km × 200 km with water depth of ~5500 m [73] (between longitude: 74–76°E and latitude: −13 and −10°S; figure 3*a*). Approximately 150 operations were carried out using Grab Sampler. The sampler with a maximum penetration depth of 15 cm and size of 50cm × 50 cm has a collection capacity of around 45 kg of wet sediment for each operation, covering a total area of ~5 m^2^ of seafloor for all these operations. Approximately 10 kg of sediments from each spot were stored in the repository (figure 3*c*), while the rest were sieved with a 200 µm mesh size immediately on the research vessel. Magnetic separation was applied to the dried >200 µm portions to isolate spherules and other magnetic materials and spherules were handpicked under a binocular microscope. The magnetic fractions primarily contained volcanogenic materials, including cosmic spherules and pumice pieces. The >200 µm non-magnetic fractions (>99% of the material) underwent heavy liquid density separation to identify cosmic spherules with weaker magnetic properties. The magnetic separate was cleaned with distilled water and dried followed by extraction of micrometeorites under binocular microscope (figure 3*d*). The presence of Australasian microtektites in three sediment cores made in the sampling area allowed to establish the chronostratigraphic position of the uppermost 15 cm. The accumulation age of this collection is estimated at ~50 000  years BP.

As of now, 1886 micrometeorites have been extracted from DSS, including 195 unmelted (figure 3b), 38 scoriaceous micrometeorites and 1653 cosmic spherules. Of the cosmic spherules, 183 are I-types, 45 are G-types and 1425 are S-types, of which the subtypes are as follows: 388 Po, 600 BO, 275 CC and 162 V-types. No CAT cosmic spherules were found in this sample. The collection also gave 195 unmelted micrometeorites by observing more than 15 000 magnetically separated particles using a binocular microscope. The extraction of micrometeorites is ongoing and samples are held under ambient environmental conditions and available upon request at the NIO, India.

### 3.3. Hot desert collections

#### 3.3.1. The Atacama Desert collection

**Figure 4.**
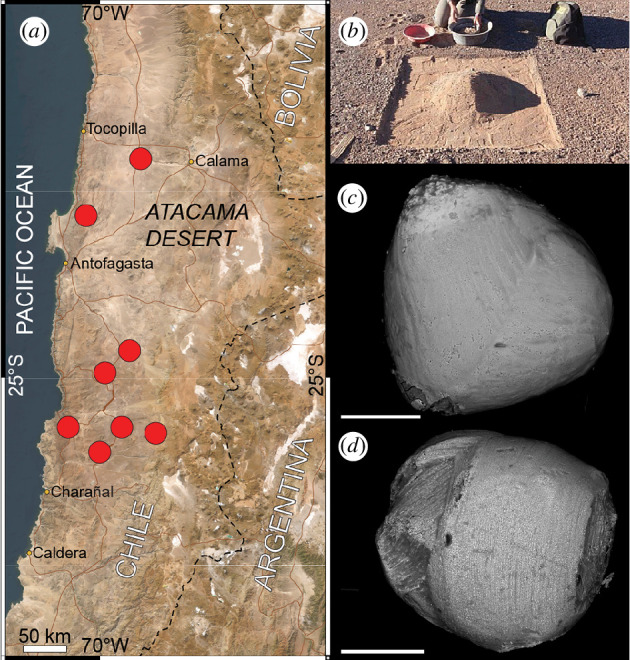
Sampling locations in the Atacama Desert in Chile (*a*). (*b*) Desert soil is being gathered before magnetic extraction and sampling. Photo credit: Jérôme Gattacceca, CEREGE. (*c*,*d*) Backscattered electron images of cosmic spherules from the Atacama Desert. The scalebars are 100 µm.

Micrometeorites were sampled from soil collected from 30 locations during expeditions organized by the Centre Européen de Recherche et d’Enseignement des Géosciences de l’Environnement (CEREGE), France, since 2006 in the Atacama Desert in Chile (figure 4*a*,*b*) [27]. Exposure ages of the sampled surface have been determined using cosmogenic nuclides to be >5 Myr [74]. Soil was first sieved (200–800 μm), and then submitted to magnetic separation. Cosmic spherules were then handpicked from the magnetic fraction under a binocular microscope, on the basis of their spherical shape and dark colour (figure 4*c*,*d*). As of now, 789 cosmic spherules have been extracted, including 129 I-types and G-types and 667 S-types (table 1). No scoriaceous and unmelted micrometeorites were identified. The extraction of micrometeorites is ongoing and the samples are held under ambient environmental conditions and available upon request at CEREGE.

It is noteworthy that other hot desert micrometeorite collections are being established by CEREGE from soil samples collected in Iran, Lybia (Dar al Gani area), Oman and Tunisia.

### 3.4. Urban collections

These recent years saw the establishment of micrometeorite collections from densely populated areas, called ‘urban’ micrometeorites [75]. These collections are largely driven by popular scientists, who have independently (with no research institution support) sought and succeeded in collecting micrometeorites, and in several cases comprise large collections.

#### 3.4.1. The Budel collection

**Figure 5.**
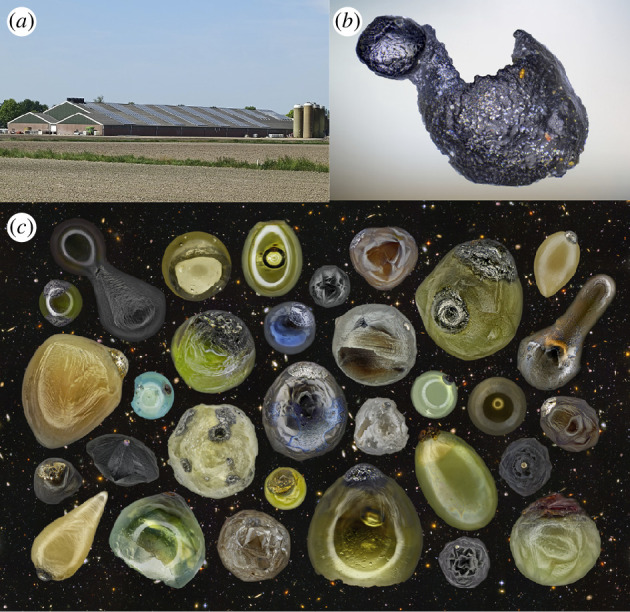
Photos of urban micrometeorites. (*a*) The rooftop of the farm from which the Budel collection was established (Photo credit: Guido Jonker, VUA). (*b*) Photomicrograph of an urban micrometeorite collected by Scott Peterson showing a metal bead that appears to be in the process of being ejected from S-type cosmic spherule. Photo credit: Scott Peterson. (*c*) Photomicrographic montage of cosmic spherules from the Project Stardust collection, showing a wide range of colours and the presence of elongated specimen. Photo credit: Jon Larsen.

The Budel collection was collected from the rooftop sediments accumulated in the gutter of a large farm in Budel, the Netherlands (figure 5*a*) [45]. The precise accumulation period of micrometeorites is 1292 days. The barn’s expansive roof area of approximately 3600 m^2^ accumulated fine aeolian dust primarily. The micrometeorite extraction process began with the initial cleaning of the samples to remove the organic matter. This included ultrasonic cleaning to enhance micrometeorite recovery by detaching them from organic particles. Subsamples underwent several sequential separation techniques to isolate micrometeorites effectively. These steps included density-based separation methods resembling gold panning to remove organic and clay/silt components, sieving using mesh sizes from 90 to 1000 μm to optimize subsequent processing, and Faultable separation to concentrate micrometeorites in spherical and subspherical fractions. Density separation further isolated micrometeorites from size fractions of 125–500 μm using a laboratory overflow centrifuge with heavy liquids. Magnetic separation was employed for particles smaller than 140 μm to recover micrometeorites. This comprehensive extraction protocol described in [45] significantly limited the sample mass while successfully isolating micrometeorites, enabling their subsequent analysis and identification.

A total of 1006 micrometeorites were found on this single rooftop, making it the largest urban micrometeorite collection from a single sampling location (table 1). These include 26 scoriaceous micrometeorites and 980 S-type cosmic spherules, of which 385 are Po, 264 are BO, 295 are CC and 36 are V-types. I-type, G-type and CAT cosmic spherules were not identified. The collection is stored under ambient environmental conditions and samples are available upon request at the Vrije Universiteit Amsterdam in the Netherlands.

#### 3.4.2. Private collections

The largest urban micrometeorite collections are private and were pioneered by Jon Larsen [75] (figure 5*c*). Particles are usually collected from accumulated sediments on flat rooftops or in the gutters of roofs. Since rooftops are generally only a few decades old, these collections cover a limited accumulation period (<50 years). Sediment samples are processed by magnetic separation either in the lab or directly on the roofs. Subsequently, collected material is washed with water and soap, dried and sieved. Cosmic spherules candidates are selected under a binocular microscope on the basis of their spherical shape, colour (black vitreous, black to grey metallic and translucent vitreous particles were selected) and their external textures. In this specific case of urban collections, similarities with spherules produced by natural processes or human activity (e.g. fly ash) often necessitate the use of SEM to confirm an extraterrestrial origin [76]. The partially melted and unmelted micrometeorites are particularly difficult to identify using a binocular microscope, because of their subrounded to angular shapes and typically black colour often shared with terrestrial particles. In addition, their rarity among micrometeorites coupled with young collection surfaces greatly reduce the probability of finding these in urban environments.

Since starting to hunt for micrometeorites in 2009, Jon Larsen has established the Project Stardust collection [77], comprising 4714 confirmed micrometeorites, of which 48 are scoriaceous and 4650 cosmic spherules (table 1). The vast majority are S-types, and additionally 2 I-types and 2 G-types. Similarly, another large urban micrometeorite collection was established by Scott Peterson [78] in Minnesota, USA, consists of 4010 confirmed micrometeorites, including 61 scoriaceous and 3932 cosmic spherules, of which 6 are I-types, 3 G-types and 3923 S-types (table 1; figure 5*b*).

## 4. Scientific potential of the micrometeorite collections

**Figure 6.**
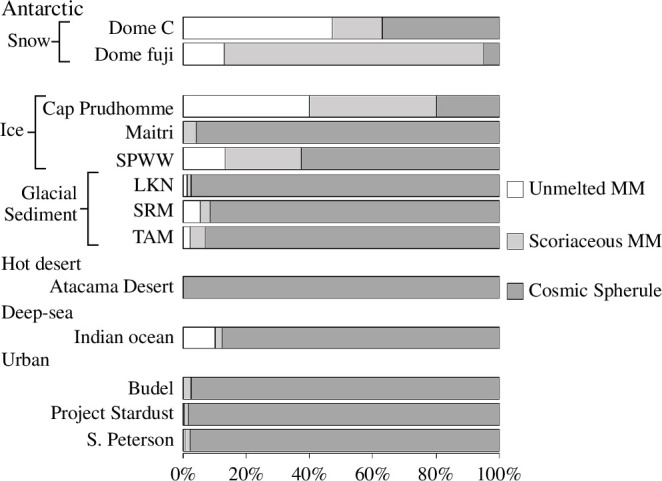
Proportions of unmelted, scoriaceous and cosmic spherules in the collections.

An essential question in planetary science pertains to the evolution of the Solar System and the inventory of extraterrestrial matter constituting it, in terms of petrophysical properties, from planetary surface, small bodies like asteroids, to cosmic dust. Studying the flux of micrometeorites is an essential step to understanding their contribution to the global inventory of extraterrestrial matter. Another essential step is the determination of the physical and chemical properties of micrometeorites, which were all but virtually unknown until the advent of electron microscopy and the establishment of the first large micrometeorite collections in the 1970s. In this section, we will show how the micrometeorite collections described above already allowed the exploration of these essential questions. Figure 6 displays classification statistics of the collections and evidences that the sampling location and/or protocols have a major impact on the relative distribution of micrometeorite group, thus affecting their potential to address specific scientific questions. Antarctica has long been considered the optimal location to search for extraterrestrial materials accreting to the Earth’s surface, because of the long-lasting cold and hyper-arid conditions inhibiting chemical weathering and the low contamination by exogenous and anthropogenic materials [46,79,80]. For these reasons, the largest and least biased micrometeorite collections are from Antarctica.

Antarctic snow and ice collections have the advantage of potentially sampling the full population of micrometeorites accreting during well-constrained time windows [81,82]. For instance, because the volume and depth of ice sampled were known, the SPWW provided a large (*n* >> 10 000) and representative sample of cosmic spherules that accreted over the last ~700 years [54]. Such a large unbiased population of cosmic spherules allowed the calculation of the first terrestrial flux for these extraterrestrial particles at 1600 ± 300 tonnes/yr [41]. Similarly, the Cap-Prudhomme collection comprises over 1 00 000 micrometeorites extracted from ice formed in pre-industrial times –50 000 years ago [49]. The potential of melting many tonnes of Antarctic ice to find large numbers of micrometeorites is further demonstrated by the recent establishment of the Maitri collection, in which almost 3000 samples have already been classified [83]. The use of a suction-based collector or metal sieves to collect and prepare sediment, however, induces mechanical stress on dust particles. The Cap-Prudhomme collection showed that large numbers of small (25–100 µm) and relatively fragile fine-grained unmelted micrometeorites can be found using this technique, however, IDP and/or IDP-like micrometeorites were probably destroyed [84]. Similarly, fine-grained micrometeorites were found in the SPWW collection, as well as the relatively tough cosmic spherules due to their igneous nature [21], while the most friable and/or smallest micrometeorites were not.

The careful sampling process used a Dome C and Dome Fuji, involving melting large quantities of ultra-clean snow and slow filtering without applying mechanical stress to the particles, prevented this limitation [37]. For this reason, the Dome C collection is considered as the least biased and representative of micrometeorites smaller than about 200 µm, allowing a determination of flux in the recent past of 5200 ± 1200 tonnes/yr when extrapolated to the 20–700 µm size range [4]. The sampling protocol also allowed the discovery of friable and small (<50 µm) fluffy fine-grained micrometeorites and the rare UCAMMs at Dome C [29,30,85,86]. The latter are samples that are unique to the Dome C and Dome Fuji collections and are characterized by an unusually high N-rich organic content (50–90%) and extreme deuterium isotopic excesses [30,87]. The discovery of CP micrometeorites at Dome Fuji [33], which are assemblages of sub-micrometre-sized minerals, demonstrated the efficiency of this sampling technique to find particularly friable and precious samples. A cometary origin was proposed for UCAMMs and CP micrometeorites based on their unique geochemical and isotopic properties, in contrast with the asteroidal nature typically associated with the micrometeorites studied so far. Both UCAMMs and CP micrometeorites exhibit assemblages of materials that are not observed in other types of extraterrestrial matter, demonstrating their importance of ultra-clean snow micrometeorite collections to probe matter in the outer Solar System, where these materials are thought to have formed. Organic matter was studied in small (<250 µm) fine-grained micrometeorites and in UCAMMs by means of Raman and Infrared (IR) spectroscopy and revealed that it mainly consists of polyaromatic carbonaceous matter with a high degree of disorder [32,87]. In several samples, the carbonaceous matter abundance was found to be larger than in carbonaceous chondrites and their mineral content exhibited differences suggesting that some of these micrometeorites may originate from distinct parent bodies than primitive carbonaceous chondrites [88].

The micrometeorite flux to planets may also have applications for their biospheres. On Earth, extraterrestrial dust may have contributed to the delivery of organic materials facilitating biosynthesis of the first living things [89,90]. Currently, the flux of micrometeorites to the Southern Oceans provides ~10% of the bioavailable Fe and this influences productivity [91]. On other planets, such as Mars, components delivered to the surface, such as organics and micronutrients, might dominate those that are bioavailable and be particularly important where the flux is higher and entry heating less extreme [92,93].

**Figure 7.**
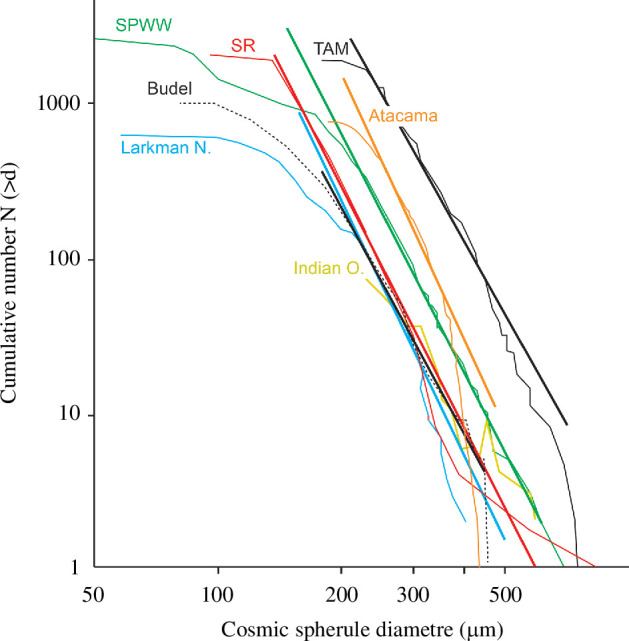
Cumulative size distributions from the SPWW [41], the Transantarctic Mountains collection (TAM; [95]), Larkman Nunatak moraine [42], Deep Sea Spherules from the Indian Ocean Collection (Indian O.; [44]), the Atacama Desert and the Sør Rondane Mountains (SR; [43]). The absolute cumulative number relates to the total number of particles collected. Inflection points are likely relate to the removal of particles by weathering or accumulation processes.

Collections from sediments collected from old and stable surfaces in Antarctica and in the Atacama Desert exhibit the longest accumulation periods (>1 Myr [27,42,63,67,94]). In particular, these collections include thousands of micrometeorites larger than 200 µm [7,26,27,95,96], which are relatively rare in ice and snow collections due to the limited accumulation periods. In the case of sediment collections from Antarctica and the Atacama Desert, statistical data from these collections have shown that in certain size ranges (table 1), the slopes of the cumulative size distribution curves of their cosmic spherules broadly match that for the most representative collection, the SPWW (figure 7). This demonstrates that these collections are representative of the flux of cosmic spherules in the larger size ranges, thus complementing snow and ice collections. Recently, the flux of micrometeorites over a long period of time (0.8–2.3 Myr) was estimated from a sampling location of the TAM collection and gave a global annual estimate of 1555 ± 753 tonnes/yr, which is consistent with the flux from the SPWW collection [40].

Rooftop collections potentially have the best-constrained accumulation windows because the ages of the collection surfaces can be known. Additionally, urban cosmic spherules usually are well-preserved, which is particularly favourable for mineralogical and chemical studies. This absence of weathering may be responsible for the occurrence of fragile features and the preservation of the Fe–Ni metal beads that frequently occur due to the immiscibility of metal in silicate melt slightly reduced in the mesosphere and that is particularly sensitive to terrestrial weathering [97,98] (figure 5*b*). Furthermore, many collection sites can be efficiently covered and micrometeorites extracted with the proper expertise [76,99]. However, little is known about potential biases such as the effect of drainage by rain water, sampling methods or cleaning of roofs, which can potentially result in the removal of almost all cosmic spherules [100]. The cumulative size distribution of cosmic spherules in the Budel collection is in good agreement with that of the SPWW collection in similar size ranges (180–450 and 200–500 µm, respectively; table 1) [45]. This suggests that biases on some rooftop collections may be efficiently minimized following the complex separation protocol established by [45]. The scientific potential of urban collections is significant due to their accessibility and potential to find extremely unusual samples (figure 5*c*).

Overall, the collections described above have so far provided important information on the flux and nature of cosmic dust accreting to Earth over the recent geological past to the present. Ultimately, it demonstrated that micrometeorites constitute the main part of extraterrestrial matter accreting to Earth. Statistical biases of specific types of collections are often counterbalanced by statistics of others (e.g. number of small versus large micrometeorites in ice/snow and sediment collections). In addition, the identification of exotic materials not observed in meteorite collections in the form of the UCAMMs and CP micrometeorites has important implications for the transfer of organic matter between asteroids and planetary surfaces. Future avenues of research include a unified flux over the whole size range, from tens of micrometres to 2 mm, to better constrain the contribution of micrometeorites to the inventory of interplanetary matter.

Identifying the parent bodies of micrometeorites is a major scientific objective and requires a good understanding of the physical and geochemical processes affecting micrometeorites during atmospheric entry and their storage in the terrestrial environment. Unmelted micrometeorites are closest to their parent material due to the low peak temperatures during atmospheric entry, particularly for the smallest particles (<<100 µm) [101]. Fine-grained micrometeorites from Antarctic collections have been shown to share mineralogical and chemical properties reminiscent of the matrices of carbonaceous chondrites dominate [22,25,84,86,102]. Conversely, coarse-grained micrometeorites may represent chondrule fragments or matrices of high petrologic type ordinary chondrites [26,103,104]. The mineralogy and chemistry of small (<50 µm) unmelted micrometeorites from the Cap-Prudhomme collection suggested that micrometeorites were compositionally homogeneous over the 25–400 µm size range [84]. As mentioned before, the presence of UCAMMs and CP micrometeorites in snow collections suggests a potential cometary contribution at <100 µm. Other Antarctic collections are shown to contain the highest number of unmelted micrometeorites (figure 6), because these are preferentially destroyed in deep-sea collections by the action of water or the sampling protocols or desert and rooftop collections focus on the more easily identifiable cosmic spherules.

Cosmic spherules represent by far the most abundant group observed in collections, except in Cap-Prudhomme (<100 µm), Dome C and Dome Fuji collections (figure 6). As such, determining their parent body is essential, albeit much more challenging due to the loss of primary mineralogical and chemical properties by melting of the precursor material [21]. Studies have shown that using the Fe/Mn ratio can provide information on the nature of cosmic spherules such as chondritic, achondritic or refractory (i.e. chondrule or calcium- and aluminium-rich inclusions) [58,59,105]. The texture of cosmic spherules has also been shown to reflect their parent material to a certain extent [27,106].

This past decade, however, the use of oxygen isotopes has proven particularly effective to identify the parent bodies of micrometeorites, and in particular cosmic spherules [7,107,108]. Large micrometeorites (>400 µm) from the TAM and Sør Rondane collections allowed the determination of oxygen isotope analyses using laser fluorination coupled with isotope ratio mass spectrometry, which offers the advantage of high precision on small volumes (<200 µg) at the expense of being totally destructive [5,7,27,63,96]. An important observation is that the contribution of carbonaceous chondritic material decreases and that of ordinary chondritic material increases with the size of micrometeorites [5]. Interestingly, an oxygen isotope study on chondritic cosmic spherules from the TAM collection has evidenced that about 10% of the cosmic spherules analysed so far exhibit ^16^O-poor compositions at odds with known meteorites, even accounting for potential isotopic fractionation effects during atmospheric entry [107]. The parent body or bodies of these ^16^O-poor cosmic spherules have yet to be identified. A recent study on ^16^O-poor unmelted micrometeorites from the TAM collection, which are thought to be related to ^16^O-poor cosmic spherules, point to CY chondrites or an unknown carbonaceous chondritic material that interacted with isotopically heavy water [109]. The presence of ^16^O-poor cosmic spherules was confirmed in the Atacama Desert collection, suggesting that these represent a non-negligible part of the micrometeorite flux about 100 µm.

It is noteworthy that of the hundreds of thousands of micrometeorite samples available in the major collections, only a fraction (i.e. «10%) have been fully characterized. The presence of the unusual ^16^O-poor micrometeorites and the recent discovery of CP micrometeorites suggest that collections may contain more mineralogical and chemical outliers associated with exotic extraterrestrial. The access to most major collections ensures that future research on micrometeorites research will result in major discoveries pertaining to the nature of interplanetary matter. Indeed, developing methods to identify and classify mineralogical and chemical outliers will further help explore the link between micrometeorites and meteorites and, as a result, the very nature of matter in the Solar System.

## 5. Potential biases affecting micrometeorite collections

We have shown above that micrometeorite collection have properties that proved useful to address specific scientific questions. However, it is essential to characterize the potential biases such as (i) often unknown accumulation period windows; (ii) the deposition environment affecting terrestrial weathering; (iii) the sampling protocol; and, (iv) surface processes affecting the concentration of micrometeorites.

Because of the removal of S-type cosmic spherules by the action of sea water, about 10% of cosmic spherules from the Indian Ocean collection are I-types, compared with ~1% in the representative SPWW collection (table 1). Additionally, about 10% of the micrometeorites recovered are unmelted, although evidences of terrestrial weathering, such as corrosion of Fe–Ni metal, are observed owing to the action of sea water. Most of the unmelted micrometeorite studied in the collection are coarse-grained, suggesting the fragile and friable fine-grained micrometeorites were preferentially lost by weathering [73].

Antarctic glacial sediment and hot desert collections have long exposure ages of up to several million years, making them minimum estimates of accumulation times. The effects of terrestrial weathering, such as the dissolution of primary minerals and replacement by secondary phases, have been defined studying the TAM and Larkman Nunatak collections [110]. While most studied micrometeorites from these two collections, along with the Sør Rondane and Atacama Desert collections [27,63], do not show evidence for advanced weathering and the rates of chemical weathering are virtually unknown, this does not preclude that some micrometeorites will be effectively destroyed well before the minimum accumulation time mentioned above, especially due to seasonal melting of potential snow cover or rare precipitations on the micrometeorite traps. Overall, the effects of terrestrial weathering may greatly vary from one sampling location to the other, for instance, due to the varying quantities of water at the sampling sites. Furthermore, determining the terrestrial age of micrometeorites of any collection, and especially cosmic spherules, is challenging because of their limited size preventing the use of conventional dating techniques such as cosmogenic nuclides or simply the resetting of geochemical properties upon melting. It is noteworthy that a study on TAM cosmic spherules has provided a rough estimate of the minimum terrestrial age of spherules by studying their magnetic properties in relation with magnetic pole reversals, thus showing that some spherules fell on Earth at least 0.78 Myr ago [111], which suggests that terrestrial weathering may be particularly inhibited in some TAM micrometeorite traps.

Biases introduced by sampling methods include the use of magnetic separation that prevents the recovery of V-type cosmic spherules; the use of mechanical preparation techniques such as sieving that will destroy the most fragile samples; micrometeorite extraction using binoculars microscopes which may be rendered difficult by clumping of particles by static electricity or high number of contaminants. While such biases may be present in all collections resulting from such sampling methods, the presence of contaminants is particularly problematic for rooftop collections, as many anthropogenic and even natural spherules look very similar to cosmic spherules. The improved collection techniques developed for the Budel collection, which include density separation, appear to effectively decrease this bias [45].

In all collections apart from DSSs, the effect of wind or potential water currents on particle concentration or removal is poorly constrained and may introduce strong accumulation biases. Surface sedimentary processes, including concentration, removal and winnowing, can introduce biases in micrometeorite collections by affecting the abundance and distribution of particles. For example, sediment traps concentrate dense particles, while winnowing affects small and low-density particles in moraines [42,47].

In summary, micrometeorite collections are extremely valuable to probe the interplanetary medium in complementarity to meteorites and asteroids, but are still influenced by various biases and uncertainties related to accumulation duration, collection methods, weathering and sedimentary processes. While improving collection methods is crucial, a good understanding of these biases is critical to establish micrometeorite collection representative of their flux to Earth.

## 6. The future of micrometeorite sampling: implications for the study of cosmic dust

Despite the systematic sampling of micrometeorites in polar regions since the 1980s, establishing the least biased collections so far, research on this subset of cosmic dust is still in its infancy. We have described the large and/or most representative collections currently available to the scientific community. However, most of these collections are far from being complete with an ongoing effort to extract more samples, namely, Dome C, Dome Fuji, Larkman Nunatak, Sør Rondane Mountains, TAM, Indian Ocean, Atacama Desert and the new Maitri collection. On the basis of the total weight of host samples collected and micrometeorite concentrations, thousands to tens of thousands of particles are expected in each of these collections. In addition, new collections are being established, for instance, the efforts of CEREGE teams to sample old surfaces of hot deserts, which can potentially yield large numbers of micrometeorites. The future of micrometeorite sampling involves completing these collections and, of equal importance, the improvement or development of new sampling protocols.

Due to their limited sizes, fossil micrometeorite collections were not included in the above sections. The effects of weathering and diagenesis severely affect the survival of micrometeorites, however, despite these limitations, cosmic spherules have been found to be frequent in the geological record [39,112–114]. Interestingly, fossil I-type cosmic spherules essentially made of magnetite may resist weathering and preserve primary geochemical information allowing their use as paleoclimatic proxies [115–117]. The establishment of future large collections of fossil micrometeorites, provided effects of diagenesis are accounted for, may provide important information on the variability of the flux of micrometeorites at Earth.

Rooftop collections are an interesting step forward as they represent the first potentially large citizen science project aiming at collecting micrometeorites. Indeed, while their scientific potential has yet to be clearly defined and their extraction is difficult and time consuming, clear advantages include the easy access to countless sampling locations in various environments, accessible guides to identify cosmic spherules [99], well-constrained accumulation periods and relatively unaltered samples. Large-scale sampling campaigns involving the scientific community and citizens have the potential to establish some of the largest collections. Improvements in collection methods [45] and assessment of potential biases (e.g. rain and wind) can provide information on the flux of micrometeorites beyond polar regions. Finally, the outreach potential of these collections already has an impact on the dissemination of micrometeorite research to a wide audience.

**Figure 8.**
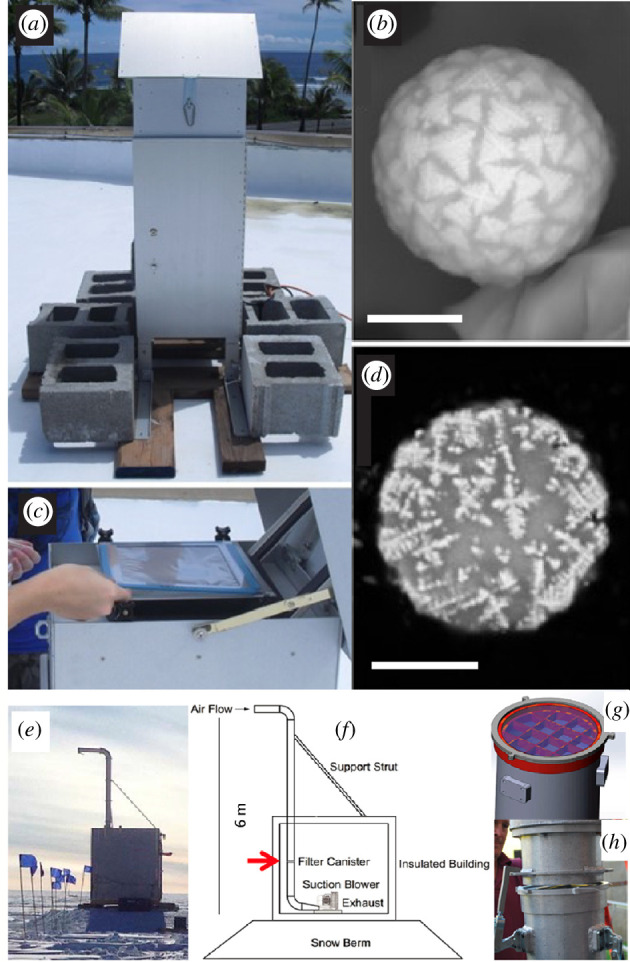
Photos of air sampling: (*a*) one of the high-volume air samplers located on the airport building at Kwajalein. (*b*) Filter change being performed. (*c*,*d*) Backscattered electron image images of exterior (*c*) and interior (*d*) of a cosmic spherule candidate identified on surveyed areas of Kwajalein filters. Scalebar is 5 µm. (*e*) Photo of building housing the air sampler at SPWW [119]. (*f*) Schematic of the collector, arrow shows the location of the filter unit in the intake pipe. (*g*) Schematic of the filter unit. (*h*) Image of the filter unit in the pipe and toggles used to clamp it shut. (*a*–*d*) Modified from [118].

A recent breakthrough in the collection of micrometeorites involves successful attempts to sample extraterrestrial particles directly from the air. Direct filtering of air or capture in the atmosphere has advantages over traditional sampling techniques. These should include low stress on the particles, minimizing contact with liquid water and a continuous record of micrometeorite and IDP fluxes and characteristics. The first attempt occurred in 2011/2012, involving a ground-based atmospheric collection method that was tested on Kwajalein Island in the Republic of the Marshall Islands [118]. This location was chosen because of its remote location over 1000 miles from the nearest continent and consistent trade winds from the northeast, offering a low anthropogenic background. Two high-volume air samplers were installed on top of an airport building and equipped with polycarbonate membrane filters (figure 8*a*,*b*). The samplers were operated over several months in 2011–2012, with filters being changed weekly. The filters were then brought back to the laboratory for study. Preliminary studies of portions of five filters (representing 5 weeks) resulted in the discovery of many cosmic spherules much smaller than 100 µm in size (figure 8*c*,*d*). This technique potentially allows determining the modern-day flux of micrometeorites by sampling a well-constrained volume of air over set periods of time. Furthermore, sporadic events such as meteor showers may be recorded, thereby pointing to specific parent bodies. Finally, cosmic spherules in a poorly studied size range (<<100 µm) may sample individual components of asteroidal and planetary materials (e.g. single crystals) and show petrographic and geochemical properties unseen in micrometeorites so far.

The second attempt at sampling cosmic dust from the air focused on IDPs at SPWW. During a 2-year experiment starting in 2016, an efficient air collector was used at SPWW to capture IDPs larger than 5 µm [119]. Housed in a dedicated building on a snow berm, the collector featured an 8 m high air intake pipe to reduce ingestion of near-surface blowing snow (figure 8*e*–*h*). In initial studies of the filters, 19 extraterrestrial particles were identified over areas representing ~0.5% of all exposed filter surfaces from 2 years. Analysing these particles involved various micro-analytical techniques, revealing valuable insights into their composition. Helium-3 analysis of filter subsamples further supported the presence of extraterrestrial material, with indications of temporal variations in the extraterrestrial small-particle flux [120].

Finally, the Dust in the Upper Stratosphere Tracking Experiment and Retrieval (DUSTER) project [121,122], an experiment attached to stratospheric balloons, has successfully collected micrometeorites in the atmosphere at the altitude of 30–40 km [123]. DUSTER includes an efficient sampling system, minimal sample manipulation, low-impact velocities to capture dust particles and strict contamination protocols. The collector consists of 13 TEM grids, allowing for the first time for particles to be exposed directly to the airflow and to adhere without the need for adhesive materials (dry collection). Five successful DUSTER launch campaigns, with the latest in 2019 and 2021 over Sweden, collected particles in the 0.1–54.0 μm size range. The particles identified so far include an I-type spherule and a porous IDP [123]. Minerals found in carbonaceous chondrites, ordinary chondrites and comets were also identified, confirming the potential of DUSTER to sample micrometeorites directly in the upper atmosphere and providing a unique window into the micron–submicron size fraction of cosmic dust.

Sampling cosmic dust directly from the air marks an important step towards drawing a comprehensive picture of micrometeorite accreting to Earth. Indeed similar air collections have also been performed at the British Antarctic Survey’s clean air sector laboratory (CASLab) at the Halley VI Research Station [124] and in Hawai’i [125].

## 7. Conclusion

We described major micrometeorite collections currently available to the scientific community, including Antarctic, DSS, hot desert and the new rooftop collections. These collections allow probing the interplanetary medium and explore the flux of cosmic dust at Earth. The first unbiased collection of micrometeorites results from the melting of large quantities of Antarctic ice. Collections from Antarctic surface snow are the most representative of the flux of micrometeorites in the ~20–200 µm size range. In addition, they yield the most friable and organic-rich UCAMMs and CP micrometeorites, which have petrological and geochemical properties not observed in other extraterrestrial materials. DSS collections show an overabundance of the iron-rich I-type cosmic spherules, allowing studies on large datasets of these spherules that are otherwise rare in other collections. Collections from Antarctic glacial sediments and Atacama Desert soil exhibit the longest continuous accumulation periods (>1 Myr), thereby giving a large number of the otherwise elusive ‘giant’ (>400 µm) micrometeorites. Although the recovery of unmelted micrometeorites remains a challenge, the new rooftop collections allow sampling of the contemporary cosmic spherule flux providing particles in a fair state of preservation.

Future micrometeorite collections should focus on fossil micrometeorites to provide clues to the flux of cosmic dust in the distant past, its variability through time, as well as act as effective paleoclimatic proxies. Three successful attempts at sampling cosmic dust directly from the air have important implications for establishing collections of the most pristine samples in an until now poorly characterized size range, typically <50 µm.

## Data Availability

The main data used in this study are provided in the paper.

## References

[1] Love SG , Brownlee DE . 1993 A direct measurement of the terrestrial mass accretion rate of cosmic dust. Science **262** , 550–553. (10.1126/science.262.5133.550) 17733236

[2] Rubin AE , Grossman JN . 2010 Meteorite and meteoroid: new comprehensive definitions. Meteorit. Planet. Sci **45** , 114–122. (10.1111/j.1945-5100.2009.01009.x)

[3] Bradley JP . 2007 1.26 - interplanetary dust particles. In In Treatise on Geochemistry (eds HD Holland , KK Turekian ), pp. 1–24. Oxford: Pergamon. (10.1016/B0-08-043751-6/01152-X)

[4] Rojas J *et al* . 2021 The micrometeorite flux at Dome C (Antarctica), monitoring the accretion of extraterrestrial dust on Earth. Earth Planet. Sci. Lett. **560** , 116794. (10.1016/j.epsl.2021.116794)

[5] Cordier C , Folco L . 2014 Oxygen isotopes in cosmic spherules and the composition of the near Earth interplanetary dust complex. Geochim. Cosmochim. Acta **146** , 18–26. (10.1016/j.gca.2014.09.038)

[6] Nesvorný D , Jenniskens P , Levison HF , Bottke WF , Vokrouhlický D , Gounelle M . 2010 Cometary origin of the zodiacal cloud and carbonaceous micrometeorites. Implications for hot debris disks. Astrophys. J. **713** , 816–836. (10.1088/0004-637X/713/2/816)

[7] Suttle MD , Dionnet Z , Franchi I , Folco L , Gibson J , Greenwood RC , Rotundi A , King A , Russell SS . 2020 Isotopic and textural analysis of giant unmelted micrometeorites – identification of new material from intensely altered 16O-poor water-rich asteroids. Earth Planet. Sci. Lett. **546** , 116444. (10.1016/j.epsl.2020.116444)

[8] Murray J , Renard MA . 1884 2. On the microscopic characters of volcanic ashes and cosmic dust, and their distribution in the deep sea deposits. Proc. R. Soc. Edinb **12** , 474–495. (10.1017/S0370164600000924)

[9] Bruun Af , Langer E , Pauly H . 1955 Magnetic particles found by raking the deep sea bottom. Deep Sea Res. **2** , 230–246. (10.1016/0146-6313(55)90027-7)

[10] Blanchard MB , Brownlee DE , Bunch TE , Hodge PW , Kyte FT . 1980 Meteoroid ablation spheres from deep-sea sediments. Earth Planet. Sci. Lett. **46** , 178–190. (10.1016/0012-821X(80)90004-7)

[11] Brownlee DE , Bates B , Schramm L . 1997 The elemental composition of stony cosmic spherules. Meteorit. Planet. Sci. **32** , 157–175. (10.1111/j.1945-5100.1997.tb01257.x)

[12] Clayton RN , Mayeda TK , Brownlee DE . 1986 Oxygen isotopes in deep-sea spherules. Earth Planet Sci. Lett. **79** , 235–240. (10.1016/0012-821X(86)90181-0)

[13] Fredriksson K . 1956 Cosmic spherules in deep-sea sediments. Nature **177** , 32–33. (10.1038/177032a0)

[14] Brownlee DE , Pilachowski LB , Hodge PW . 1979 Meteorite Mining on the Ocean Floor. In Lunar and Planetary Science Conference. pp. 157–158

[15] Goresy AE . 1968 Electron microprobe analysis and ore microscopic study of magnetic spherules and grains collected from the Greenland ice. Contr. Mineral. and Petrol. **17** , 331–346. (10.1007/BF00380743)

[16] Maurette M , Jéhanno C , Robin E , Hammer C . 1987 Characteristics and mass distribution of extraterrestrial dust from the Greenland ice cap. Nature **328** , 699–702. (10.1038/328699a0)

[17] Iwata N , Imae N . 2002 Antarctic micrometeorite collection at a bare ice region near Syowa Station by JARE-41 in 2000. Antarct. meteor. res. **15** , 25.

[18] Yada T , Kojima H . 2000 The collection of micrometeorites in the Yamato Meteorite Ice Field of Antarctica in 1998. Antarct. meteor. res. **13** , 9.

[19] Harvey RP , Maurette M . 1991 The origin and significance of cosmic dust from the Walcott Névé, Antarctica. Lunar and Planetary Science Conference Proceedings **21** , 569–578.

[20] Badjukov DD , Raitala J . 2003 Micrometeorites from the northern ice cap of the Novaya Zemlya archipelago, Russia: the first occurrence. Meteorit. Planet. Sci. **38** , 329–340. (10.1111/j.1945-5100.2003.tb00269.x)

[21] Genge MJ , Engrand C , Gounelle M , Taylor S . 2008 The classification of micrometeorites. Meteorit. Planet. Sci. **43** , 497–515. (10.1111/j.1945-5100.2008.tb00668.x)

[22] Engrand C , Maurette M . 1998 Carbonaceous micrometeorites from Antarctica. Meteorit. Planet. Sci. **33** , 565–580. (10.1111/j.1945-5100.1998.tb01665.x) 11543069

[23] Suttle MD , Genge MJ , Folco L , Russell SS . 2017 The thermal decomposition of fine-grained micrometeorites, observations from mid-IR spectroscopy. Geochim. Cosmochim. Acta **206** , 112–136. (10.1016/j.gca.2017.03.002)

[24] Suttle MD , Genge MJ , Folco L , Van Ginneken M , Lin Q , Russell SS , Najorka J . 2019 The atmospheric entry of fine‐grained micrometeorites: The role of volatile gases in heating and fragmentation. Meteorit. Planet. Sci. **54** , 503–520. (10.1111/maps.13220)

[25] Genge MJ , Grady MM , Hutchison R . 1997 The textures and compositions of fine-grained Antarctic micrometeorites: Implications for comparisons with meteorites. Geochim. Cosmochim. Acta **61** , 5149–5162. (10.1016/S0016-7037(97)00308-6)

[26] Van Ginneken M , Folco L , Cordier C , Rochette P . 2012 Chondritic micrometeorites from the Transantarctic Mountains. Meteorit. Planet. Sci. **47** , 228–247. (10.1111/j.1945-5100.2011.01322.x) PMC2583132 19011091

[27] van Ginneken M , Gattacceca J , Rochette P , Sonzogni C , Alexandre A , Vidal V , Genge MJ . 2017 The parent body controls on cosmic spherule texture: Evidence from the oxygen isotopic compositions of large micrometeorites. Geochim. Cosmochim. Acta **212** , 196–210. (10.1016/j.gca.2017.05.008)

[28] Gounelle M , Chaussidon M , Morbidelli A , Barrat JA , Engrand C , Zolensky ME , McKeegan KD . 2009 A unique basaltic micrometeorite expands the inventory of solar system planetary crusts. Proc. Natl. Acad. Sci. USA **106** , 6904–6909. (10.1073/pnas.0900328106) 19366660 PMC2678474

[29] Dartois E *et al* . 2013 UltraCarbonaceous Antarctic micrometeorites, probing the Solar System beyond the nitrogen snow-line. Icarus **224** , 243–252. (10.1016/j.icarus.2013.03.002)

[30] Duprat J *et al* . 2010 Extreme deuterium excesses in ultracarbonaceous micrometeorites from central Antarctic snow. Science (1979) **328** , 742–745. (10.1126/science.1184832) 20448182

[31] Yabuta H *et al* . 2017 Formation of an ultracarbonaceous Antarctic micrometeorite through minimal aqueous alteration in a small porous icy body. Geochim. Cosmochim. Acta **214** , 172–190. (10.1016/j.gca.2017.06.047)

[32] Dobrică E , Engrand C , Quirico E , Montagnac G , Duprat J . 2011 Raman characterization of carbonaceous matter in CONCORDIA Antarctic micrometeorites. Meteorit. Planet. Sci. **46** , 1363–1375. (10.1111/j.1945-5100.2011.01235.x)

[33] Noguchi T *et al* . 2015 Cometary dust in Antarctic ice and snow: past and present chondritic porous micrometeorites preserved on the Earth’s surface. Earth Planet Sci. Lett. **410** , 1–11. (10.1016/j.epsl.2014.11.012)

[34] Matsuoka K *et al* . 2021 Quantarctica, an integrated mapping environment for Antarctica, the Southern Ocean, and sub-Antarctic islands. Environ. Model. Softw. **140** , 105015. (10.1016/j.envsoft.2021.105015)

[35] Toppani A , Libourel G . 2003 Factors controlling compositions of cosmic spinels: application to atmospheric entry conditions of meteoritic materials. Geochim. Cosmochim. Acta **67** , 4621–4638. (10.1016/S0016-7037(03)00383-1)

[36] Genge MJ . 2006 Igneous rims on micrometeorites. Geochim. Cosmochim. Acta **70** , 2603–2621. (10.1016/j.gca.2006.02.005)

[37] Duprat J , Engrand C , Maurette M , Kurat G , Gounelle M , Hammer C . 2007 Micrometeorites from Central Antarctic snow: The CONCORDIA collection. Adv. Space Res. **39** , 605–611. (10.1016/j.asr.2006.05.029)

[38] Genge MJ , Davies B , Suttle MD , van Ginneken M , Tomkins AG . 2017 The mineralogy and petrology of I-type cosmic spherules: implications for their sources, origins and identification in sedimentary rocks. Geochim. Cosmochim. Acta **218** , 167–200. (10.1016/j.gca.2017.09.004)

[39] Taylor S , Brownlee DE . 1991 Cosmic spherules in the geologic record. Meteoritics **26** , 203–211. (10.1111/j.1945-5100.1991.tb01040.x)

[40] Suttle MD , Folco L . 2020 The extraterrestrial dust flux: size distribution and mass contribution estimates inferred from the Transantarctic Mountains (TAM) micrometeorite collection. J. Geophys. Res. Planets **125** , e2019JE006241. (10.1029/2019JE006241)

[41] Taylor S , Lever JH , Harvey RP . 1998 Accretion rate of cosmic spherules measured at the South Pole. Nature **392** , 899–903. (10.1038/31894) 9582069

[42] Genge MJ , van Ginneken M , Suttle MD , Harvey RP . 2018 Accumulation mechanisms of micrometeorites in an ancient supraglacial moraine at Larkman Nunatak, Antarctica. Meteorit. Planet. Sci. **53** , 2051–2066. (10.1111/maps.13107)

[43] van Maldeghem F , van Ginneken M , Soens B , Kaufmann F , Lampe S , Krämer Ruggiu L , Hecht L , Claeys P , Goderis S . 2023 Geochemical characterization of scoriaceous and unmelted micrometeorites from the Sør Rondane Mountains, East Antarctica: links to chondritic parent bodies and the effects of alteration. Geochim. Cosmochim. Acta **354** , 88–108. (10.1016/j.gca.2023.06.002)

[44] Prasad MS , Rudraswami NG , Panda DK. 2013 Micrometeorite flux on Earth during the last ~50 000 years. J. Geophys. Res. Planets. 118, 2381–2399. (10.1002/2013JE004460)

[45] Jonker G , van Elsas R , van der Lubbe JHJL , van Westrenen W . 2023 Improved collection of rooftop micrometeorites through optimized extraction methods: the Budel collection. Meteorit. Planet. Sci. **58** , 463–479. (10.1111/maps.13966)

[46] Harvey R . 2003 The origin and significance of Antarctic meteorites. Geochemistry **63** , 93–147. (10.1078/0009-2819-00031)

[47] Genge MJ , van Ginneken M , Suttle MD . 2020 Micrometeorites: insights into the flux, sources and atmospheric entry of extraterrestrial dust at Earth. Planet Space Sci. **187** , 104900. (10.1016/j.pss.2020.104900)

[48] Maurette M , Olinger C , Michel-Levy MC , Kurat G , Pourchet M , Brandstätter F , Bourot-Denise M . 1991 A collection of diverse micrometeorites recovered from 100 tonnes of Antarctic blue ice. Nature **351** , 44–47. (10.1038/351044a0)

[49] Maurette M , Immel G , Hammer C , Harvey R , Kurat G , Taylor S . 1994 Collection and curation of IDPs from the Greenland and Antarctic ice sheets. AIP Conf. Proc. **310** , 277–290. (10.1063/1.46516)

[50] Gounelle M , Maurette M , Engrand C , Brandstätter F , Kurat G . 1999 Mineralogy of the 1998 Astrolabe Antarctic micrometeorite collection. Meteorit. Planet. Sci. Supp. **34** , A46.

[51] Engrand C . 2008 Micrométéorites Concordia: des Neiges Antarctiques aux Glaces Cométaires. In diplôme d’habilitation à diriger des recherches. See https://theses.hal.science/tel-00549129/document.

[52] Noguchi T , Yamaguchi A , Imae N . 2022 Accretion rate of Antarctic micrometeorites stored in surface snow near Dome Fuji Station. In Royal Astronomical Society virtual meeting: Sources and inventory of cosmic dust: From space to the Earth’s surface. London: Royal Astronomical Society.

[53] Kameda T , Motoyama H , Fujita S , Takahashi S . 2008 Temporal and spatial variability of surface mass balance at Dome Fuji, East Antarctica, by the stake method from 1995 to 2006. J. Glaciol. **54** , 107–116. (10.3189/002214308784409062)

[54] Taylor S , Lever JH , Harvey RP . 2000 Numbers, types, and compositions of an unbiased collection of cosmic spherules. Meteorit. Planet Sci. **35** , 651–666. (10.1111/j.1945-5100.2000.tb01450.x)

[55] Taylor S , Lever J , Harvey R . 1995 A Micrometeorite Collector for the South Pole Water Well. In Lunar and Planetary Science Conference. p. 1401

[56] Kuiniven KC , Koci BR , Holdworth GW , Gow AJ . 1982 South Pole ice core drilling, 1981-1982. Antarct. J. US **17** , 89–91.

[57] Taylor S , Lever JH , Govoni J . 2001 A second collection of micrometeorites from the south pole water well. In Lunar and Planetary Science Conference. p. 1914

[58] Taylor S , Herzog GF , Delaney JS . 2007 Crumbs from the crust of Vesta: Achondritic cosmic spherules from the South Pole water well. Meteorit. Planet. Sci. **42** , 223–233. (10.1111/j.1945-5100.2007.tb00229.x)

[59] Cordier C , Folco L , Taylor S . 2011 Vestoid cosmic spherules from the South Pole water well and transantarctic mountains (Antarctica): a major and trace element study. Geochim. Cosmochim. Acta **75** , 1199–1215. (10.1016/j.gca.2010.11.024)

[60] Taylor S , Lindsay FN , Delaney JS , Herzog GF . 2013 Micrometeorite SP-F88: A Lunar or an Angrite? In 44nd Lunar and Planetary Science Conference. p. 1517

[61] Taylor S , Alexander CMO , Wengert S . 2008 Rare Micrometeorites from the South Pole, Antarctica. In 39th Annual Lunar and Planetary Science Conference. p. 1628

[62] Van Ginneken M , Genge MJ , Harvey RP . 2018 A new type of highly-vaporized microtektite from the Transantarctic Mountains. Geochim. Cosmochim. Acta **228** , 81–94. (10.1016/j.gca.2018.02.041)

[63] Goderis S *et al* . 2020 Cosmic spherules from Widerøefjellet, Sør Rondane Mountains (East Antarctica). Geochim. Cosmochim. Acta **270** , 112–143. (10.1016/j.gca.2019.11.016)

[64] Suganuma Y , Miura H , Zondervan A , Okuno J . 2014 East Antarctic deglaciation and the link to global cooling during the Quaternary: evidence from glacial geomorphology and 10Be surface exposure dating of the Sør Rondane Mountains, Dronning Maud Land. Quat. Sci. Rev. **97** , 102–120. (10.1016/j.quascirev.2014.05.007)

[65] Soens B , van Ginneken M , Chernonozhkin S , Slotte N , Debaille V , Vanhaecke F , Terryn H , Claeys P , Goderis S . 2021 Australasian microtektites across the Antarctic continent: Evidence from the Sør Rondane Mountain range (East Antarctica). Geosci. Front. **12** , 101153. (10.1016/j.gsf.2021.101153)

[66] Van Ginneken M *et al* . 2021 A large meteoritic event over Antarctica ca. 430 ka ago inferred from chondritic spherules from the Sør Rondane Mountains. Sci. Adv. **7** , eabc1008. (10.1126/sciadv.abc1008) 33789890 PMC8011977

[67] Rochette P , Folco L , Suavet C , van Ginneken M , Gattacceca J , Perchiazzi N , Braucher R , Harvey RP . 2008 Micrometeorites from the transantarctic mountains. Proc. Natl. Acad. Sci. USA **105** , 18206–18211. (10.1073/pnas.0806049105) 19011091 PMC2583132

[68] Welten KC , Folco L , Nishiizumi K , Caffee MW , Grimberg A , Meier MMM , Kober F . 2008 Meteoritic and bedrock constraints on the glacial history of Frontier Mountain in northern Victoria Land, Antarctica. Earth Planet. Sci. Lett. **270** , 308–315. (10.1016/j.epsl.2008.03.052)

[69] Folco L , Rochette P , Perchiazzi N , D’Orazio M , Laurenzi MA , Tiepolo M . 2008 Microtektites from Victoria land transantarctic mountains. Geology **36** , 291–294. (10.1130/G24528A.1)

[70] van Ginneken M , Folco L , Perchiazzi N , Rochette P , Bland PA . 2010 Meteoritic ablation debris from the Transantarctic Mountains: Evidence for a Tunguska-like impact over Antarctica ca. 480ka ago. Earth Planet Sci. Lett. **293** , 104–113. (10.1016/j.epsl.2010.02.028)

[71] Van Ginneken M , Suavet C , Cordier C , Folco L , Rochette P , Sonzogni C , Perchiazzi N . 2012 Oxygen isotope composition of meteoritic ablation debris from the Transantarctic Mountains: constraining the parent body and implications for the impact scenario. Meteorit. Planet. Sci. **47** , 1738–1747. (10.1111/maps.12011)

[72] Brownlee DE 1985 Cosmic dust - Collection and research. Annu. Rev. Earth Planet. Sci. 13, 147–173. (10.1146/annurev.ea.13.050185.001051)

[73] Prasad MS , Rudraswami NG , de Araujo AA , Khedekar VD . 2018 Characterisation, sources and flux of unmelted micrometeorites on earth during the last ~50 000 years. Sci. Rep. **8** , 8887. (10.1038/s41598-018-27158-x) 29891909 PMC5995856

[74] Hutzler A *et al* . 2016 Description of a very dense meteorite collection area in western Atacama: Insight into the long-term composition of the meteorite flux to Earth. Meteorit. Planet Sci. 51, 468–482. (10.1111/maps.12607)

[75] Genge MJ , Larsen J , Van Ginneken M , Suttle MD . 2017 An urban collection of modern-day large micrometeorites: Evidence for variations in the extraterrestrial dust flux through the Quaternary. Geology **45** , 119–122. (10.1130/G38352.1)

[76] Larsen J . 2016 In search of Stardust. Oslo, Norway: Arthouse DGB/Kunstbokforlaget.

[77] Larsen J . 2022 Project Stardust. See https://projectstardust.xyz

[78] Bland PA , Zolensky ME , Benedix GK , Sephton MA . Weathering of Chondritic meteorites. In Meteorites and the early solar system II (eds DS Lauretta , HY McSween ), pp. 853–867. Tucson, Arizona: University of Arizona Press. (10.2307/j.ctv1v7zdmm)

[79] Jull AJT . 2001 In Terrestrial Ages of Meteorites (eds B Peucker-Ehrenbrink , B Schmitz ), pp. 241–266. Boston, MA: Springer US. (10.1007/978-1-4419-8694-8_14)

[80] Bland PA , Zolensky ME , Benedix GK , Sephton MA . Weathering of Chondritic meteorites. In Meteorites and the early solar system II (eds D Lauretta , HY McSween ), p. 853, (10.2307/j.ctv1v7zdmm)

[81] Taylor S , Lever JH . 2001 Seeking unbiased collections of modern and ancient micrometeorites. In Accretion of Extraterrestrial Matter Throughout Earth’s History (ed. B Schmitz ), pp. 205–219, Boston, MA: Springer US. (10.1007/978-1-4419-8694-8)

[82] Folco L , Cordier C . 2015 Micrometeorites. In Planetary Mineralogy. European Mineralogical Union. (10.1180/EMU-notes.15)

[83] Rudraswami NG , Fernandes D , Pandey M . 2020 Probing the nature of extraterrestrial dust reaching the Earth’s surface collected from the Maitri station, Antarctica. Meteorit. Planet. Sci. **55** , 2256–2266. (10.1111/maps.13574)

[84] Gounelle M , Engrand C , Maurette M , Kurat G , McKeegan KD , Brandstätter F . 2005 Small Antarctic micrometeorites: a mineralogical and in situ oxygen isotope study. Meteorit. Planet. Sci. **40** , 917–932. (10.1111/j.1945-5100.2005.tb00163.x)

[85] Dobrică E , Engrand C , Leroux H , Rouzaud JN , Duprat J . 2012 Transmission electron microscopy of CONCORDIA UltraCarbonaceous Antarctic MicroMeteorites (UCAMMs): mineralogical properties. Geochim. Cosmochim. Acta **76** , 68–82. (10.1016/j.gca.2011.10.025)

[86] Dobricǎ E , Engrand C , Duprat J , Gounelle M , Leroux H , Quirico E , Rouzaud J ‐N . 2009 Connection between micrometeorites and Wild 2 particles: from Antarctic snow to cometary ices. Meteorit. Planet. Sci. **44** , 1643–1661. (10.1111/j.1945-5100.2009.tb01196.x)

[87] Dartois E , Engrand C , Duprat J , Godard M , Charon E , Delauche L , Sandt C , Borondics F . 2018 Dome C ultracarbonaceous Antarctic micrometeorites - Infrared and Raman fingerprints. A&A **609** , A65. (10.1051/0004-6361/201731322)

[88] Battandier M , Bonal L , Quirico E , Beck P , Engrand C , Duprat J , Dartois E . 2018 Characterization of the organic matter and hydration state of Antarctic micrometeorites: a reservoir distinct from carbonaceous chondrites. Icarus **306** , 74–93. (10.1016/j.icarus.2018.02.002)

[89] Maurette M . 1998 Carbonaceous micrometeorites and the origin of life. Orig. Life Evol. Biosph. **28** , 385–412. (10.1023/A:1006589819844) 10357645

[90] Maurette M , Brack A , Kurat G , Perreau M , Engrand C . 1995 Were micrometeorites a source of prebiotic molecules on the early Earth? Adv. Space Res. **15** , 113–126. (10.1016/s0273-1177(99)80071-4) 11539212

[91] Rudraswami NG , Pandey M , Genge MJ , Fernandes D . 2021 Extraterrestrial dust as a source of bioavailable iron contributing to the ocean for driving primary productivity. Meteorit. Planet. Sci. **56** , 2175–2190. (10.1111/maps.13764)

[92] Tomkins AG , Genge MJ , Tait AW , Alkemade SL , Langendam AD , Perry PP , Wilson S . 2019 High survivability of micrometeorites on mars: sites with enhanced availability of limiting nutrients. J. Geophys. Res. Planets. **124** , 1802–1818. (10.1029/2019JE006005)

[93] Wilson AP , Genge MJ , Krzesińska AM , Tomkins AG . 2019 Atmospheric entry heating of micrometeorites at Earth and Mars: implications for the survival of organics. Meteorit. Planet. Sci. **54** , 1–19. (10.1111/maps.13360)

[94] Dunai TJ , González López GAG , Juez-Larré J . 2005 Oligocene–Miocene age of aridity in the Atacama Desert revealed by exposure dating of erosion-sensitive landforms. Geology **33** , 321–324. (10.1130/G21184.1)

[95] Suavet C , Rochette P , Kars M , Gattacceca J , Folco L , Harvey RP . 2009 Statistical properties of the Transantarctic Mountains (TAM) micrometeorite collection. Polar Sci. **3** , 100–109. (10.1016/j.polar.2009.06.003)

[96] Suavet C , Cordier C , Rochette P , Folco L , Gattacceca J , Sonzogni C , Damphoffer D . 2011 Ordinary chondrite-related giant (>800μm) cosmic spherules from the Transantarctic Mountains, Antarctica. Geochim. Cosmochim. Acta **75** , 6200–6210. (10.1016/j.gca.2011.07.034)

[97] Genge MJ , Grady MM . 1998 Melted micrometeorites from Antarctic ice with evidence for the separation of immiscible Fe‐Ni‐S liquids during entry heating. Meteorit. Planet. Sci. **33** , 425–434. (10.1111/j.1945-5100.1998.tb01647.x)

[98] van Ginneken M , Genge MJ , Folco L , Harvey RP . 2016 The weathering of micrometeorites from the Transantarctic Mountains. Geochim. Cosmochim. Acta **179** , 1–31. (10.1016/j.gca.2015.11.045)

[99] Larsen J . 2021 How to find Stardust. Oslo, Norway: Arthouse DGB/Kunstbokforlaget.

[100] Suttle MD , Hasse T , Hecht L . 2021 Evaluating urban micrometeorites as a research resource—a large population collected from A single rooftop. Meteorit. Planet. Sci. **56** , 1531–1555. (10.1111/maps.13712)

[101] Love SG , Brownlee DE . 1991 Heating and thermal transformation of micrometeoroids entering the Earth’s atmosphere. Icarus **89** , 26–43. (10.1016/0019-1035(91)90085-8)

[102] Kurat G , Koeberl C , Presper T , Brandstätter F , Maurette M . 1994 Petrology and geochemistry of Antarctic micrometeorites. Geochim. Cosmochim. Acta **58** , 3879–3904. (10.1016/0016-7037(94)90369-7)

[103] Genge MJ . 2008 Koronis asteroid dust within Antarctic ice. Geology **36** , 687–690. (10.1130/G24493A.1)

[104] Genge MJ , Gileski A , Grady MM . 2005 Chondrules in Antarctic micrometeorites. Meteorit. Planet. Sci. **40** , 225–238. (10.1111/j.1945-5100.2005.tb00377.x)

[105] Soens B , Chernonozhkin SM , González de Vega C , Vanhaecke F , van Ginneken M , Claeys P , Goderis S . 2022 Characterization of achondritic cosmic spherules from the Widerøefjellet micrometeorite collection (Sør Rondane Mountains, East Antarctica). Geochim. Cosmochim. Acta **325** , 106–128. (10.1016/j.gca.2022.03.029)

[106] Taylor S , Matrajt G , Guan Y . 2012 Fine‐grained precursors dominate the micrometeorite flux. Meteorit. Planet. Sci. **47** , 550–564. (10.1111/j.1945-5100.2011.01292.x)

[107] Suavet C , Alexandre A , Franchi IA , Gattacceca J , Sonzogni C , Greenwood RC , Folco L , Rochette P . 2010 Identification of the parent bodies of micrometeorites with high-precision oxygen isotope ratios. Earth Planet. Sci. Lett. **293** , 313–320. (10.1016/j.epsl.2010.02.046)

[108] Rudraswami NG , Prasad MS , Nagashima K , Jones RH . 2015 Oxygen isotopic composition of relict olivine grains in cosmic spherules: Links to chondrules from carbonaceous chondrites. Geochim. Cosmochim. Acta **164** , 53–70. (10.1016/j.gca.2015.05.004)

[109] Suttle MD , Folco L , Dionnet Z , Van Ginneken M , Di Rocco T , Pack A , Scheel M , Rotundi A . 2022 Isotopically heavy micrometeorites—fragments of CY chondrite or a new hydrous parent body? J. Geophys. Res. Planets. **127** , e2021JE007154. (10.1029/2021JE007154)

[110] van Ginneken M , Genge MJ , Folco L , Harvey RP . 2016 The weathering of micrometeorites from the Transantarctic Mountains. Geochim. Cosmochim. Acta **179** , 1–31. (10.1016/j.gca.2015.11.045)

[111] Suavet C , Gattacceca J , Rochette P , Folco L . 2011 Constraining the terrestrial age of micrometeorites using their record of the Earth’s magnetic field polarity. Geology **39** , 123–126. (10.1130/G31655.1)

[112] Suttle MD , Genge MJ . 2017 Diagenetically altered fossil micrometeorites suggest cosmic dust is common in the geological record. Earth Planet. Sci. Lett. **476** , 132–142. (10.1016/j.epsl.2017.07.052)

[113] Suttle MD *et al* . 2023 Fossil micrometeorites from Monte dei Corvi: searching for dust from the Veritas asteroid family and the utility of micrometeorites as a palaeoclimate proxy. Geochim. Cosmochim. Acta **355** , 75–88. (10.1016/j.gca.2023.06.027)

[114] Voldman GG , Genge MJ , Albanesi GL , Barnes CR , Ortega G . 2013 Cosmic spherules from the Ordovician of Argentina. Geol. J. **48** , 222–235. (10.1002/gj.2418)

[115] Tomkins AG , Bowlt L , Genge M , Wilson SA , Brand HEA , Wykes JL . 2016 Ancient micrometeorites suggestive of an oxygen-rich Archaean upper atmosphere. Nature **533** , 235–238. (10.1038/nature17678) 27172047

[116] Fischer MB , Oeser M , Weyer S , Folco L , Peters STM , Zahnow F , Pack A . 2021 I‐Type cosmic spherules as proxy for the Δ′17O of the atmosphere—a calibration with quaternary air. Paleoceanogr. Paleoclimatol. **36** , e2020PA004159. (10.1029/2020PA004159)

[117] Pack A , Höweling A , Hezel DC , Stefanak MT , Beck AK , Peters STM , Sengupta S , Herwartz D , Folco L . 2017 Tracing the oxygen isotope composition of the upper Earth’s atmosphere using cosmic spherules. Nat. Commun. **8** , 15702. (10.1038/ncomms15702) 28569769 PMC5461487

[118] Wozniakiewicz PJ , Alesbrook LS , Bradley JP , Ishii HA , Price MC , Zolensky ME , Brownlee DE , Van Ginneken M , Genge MJ . 2024 Atmospheric collection of extraterrestrial dust at the earth’s surface in the mid-Pacific. Accepted in Meteorit Planet. Sci.

[119] Taylor S *et al* . 2020 Sampling interplanetary dust from Antarctic air. Meteorit. &. Planetary Sci. **55** , 1128–1145. (10.1111/maps.13483)

[120] Farley KA , Taylor S , Treffkorn J , Lever JH , Gow AL . 2021 3He flux obtained from South Pole air and snow‐ice and its connection to interplanetary dust particles. Meteorit. Planet. Sci. **56** , 1988–2001. (10.1111/maps.13759)

[121] Della Corte V , Rietmeijer FJM , Rotundi A , Ferrari M . 2014 Introducing a new stratospheric dust-collecting system with potential use for upper atmospheric microbiology investigations. Astrobiology **14** , 694–705. (10.1089/ast.2014.1167) 25046407 PMC4126274

[122] Della Corte V *et al* . 2012 In situ collection of refractory dust in the upper stratosphere: the DUSTER facility. Space Sci. Rev. **169** , 159–180. (10.1007/s11214-012-9918-9)

[123] Musolino A , Della Corte V , Rotundi A , Dionnet Z , Folco L , Liuzzi V , Ferretti S . 2022 Dust in the upper stratosphere tracking experiment and retrieval: exploring the dust reservoir of the upper stratosphere through balloons - HEMERA campaigns preliminary results. In 44th COSPAR Scientific Assembly. Held 16-24 July p. 155,

[124] Alesbrook LS , Wozniakiewicz PJ , Jones AE , Price MC , Ishii HA , Bradley JP , Brough N . 2017 Atmospheric collection of extraterrestrial dust at the Halley Research Station, Antarctica. In 48th Annual Lunar and Planetary Science Conference. p. 1805

[125] Ishii HA , Wozniakiewicz PJ , Bradley JP , Farley K , Martinsen M . 2017 Extraterrestrial dust collection at Mauna Loa Observatory, Hawaii. In 48th Annual Lunar and Planetary Science Conference. p. 1141

